# Lipid clearance and amyloid formation by serum amyloid A: exploring the links between beneficial and pathologic actions of an enigmatic protein

**DOI:** 10.1016/j.jlr.2023.100429

**Published:** 2023-08-19

**Authors:** Shobini Jayaraman, Angela Urdaneta, Esther Bullitt, Marcus Fändrich, Olga Gursky

**Affiliations:** 1 Department of Pharmacology, Physiology & Biophysics, Chobanian and Avedisian School of Medicine, Boston University, Boston, MA, USA; 2 Institute of Protein Biochemistry, Ulm University, Ulm, Germany

**Keywords:** inflammation and systemic amyloidosis, secretory phospholipase A2, heparan sulfate mimetic, scavenger receptor CD36, lipoproteins

## Abstract

Serum amyloid A (SAA) is named after a life-threatening disease, yet this small evolutionarily conserved protein must have played a vital role in host defense. Most circulating SAA binds plasma lipoproteins and modulates their metabolism. However, this hardly justifies the rapid and dramatic SAA upregulation in inflammation, which is concomitant with upregulation of secretory phospholipase A_2_ (sPLA_2_). We proposed that these proteins synergistically clear cell membrane debris from the sites of injury. The present study uses biochemical and biophysical approaches to further explore the beneficial function of SAA and its potential links to amyloid formation. We show that murine and human SAA1 are powerful detergents that solubilize diverse lipids, including mammalian biomembranes, converting them into lipoprotein-size nanoparticles. These nanoparticles provide ligands for cell receptors, such as scavenger receptor CD36 or heparin/heparan sulfate, act as substrates of sPLA_2_, and sequester toxic products of sPLA_2._ Together, these functions enable SAA to rapidly clear unprotected lipids. SAA can also adsorb, without remodeling, to lipoprotein-size nanoparticles such as exosomal liposomes, which are proxies for lipoproteins. SAA in complexes with zwitterionic phospholipids stabilizes α-helices, while SAA in complexes containing anionic lipids or micelle-forming sPLA_2_ products forms metastable β-sheet–rich species that readily aggregate to form amyloid. Consequently, the synergy between SAA and sPLA_2_ extends from the beneficial lipid clearance to the pathologic amyloid formation. Furthermore, we show that lipid composition alters SAA conformation and thereby can influence the metabolic fate of SAA–lipid complexes, including their proamyloidogenic and proatherogenic binding to heparan sulfate.

Serum amyloid A (SAA) (12 kDa) is a family of highly evolutionarily conserved proteins that plays a vital yet enigmatic role in host defense and is best known for its association with disease. SAA is an acute phase response protein that is elevated in response to inflammation, infection, and tissue damage ((1, 2, 3, 4, 5, 6) and references therein). Although systemic SAA levels provide biomarkers of disease and readouts for treatment, whether they contribute to disease is unclear (3, 4). Furthermore, SAA is a protein precursor of amyloid A (AA) amyloidosis, the major systemic amyloid disease in animals and a life-threatening complication of chronic inflammation in humans, which can cause kidney damage in a subset of patients with chronic infections, autoimmune disorders, and certain cancers (6, 7). SAA is also found in atherosclerotic lesions and other lipoprotein-derived arterial deposits (8, 9), and elevated SAA is a marker and a causal factor for atherosclerosis in mice and men (10). Binding of SAA to plasma lipoproteins and to heparan sulfate (HS) proteoglycans (PGs) may contribute to the causal link between inflammation and cardiovascular disease (10, 11), as lipoprotein retention by arterial HSPGs is an early trigger of atherosclerosis (12). Importantly, SAA binding to extracellular HSPGs augments the development of AA amyloidosis and is an established therapeutic target in AA amyloidosis (1, 13). However, blocking this binding by a small-molecule HS mimetic, eprodisate (14), failed to show efficacy in clinical trials and is addressed in this study.

In acute inflammation, infection, or injury, plasma levels of SAA isoforms 1 and 2 raise rapidly but transiently by nearly 1,000-fold, while in chronic inflammation SAA is moderately but persistently elevated (3, 4). The biological advantage conferred by elevated SAA is perplexing; the consensus is that SAA is involved in inflammation control, immune response, and lipid transport, yet the details of these and other SAA functions are subjects of debate (3, 4, 6, 15). SAA in humans and mice is synthesized by the liver and by other tissues locally at the inflammation sites. Most circulating SAA binds to plasma HDLs and alters their metabolism by interacting with various cell receptors including CD36, LOX1, RAGE, etc. (reviewed in (3, 4, 6, 15)). Through these and other interactions SAA has been proposed to reroute HDL cholesterol transport for cell repair (1), activate innate immunity, modulate the onset and the resolution of inflammation, mediate retinol transport (16), and perform various other functions (2, 3, 5, 6, 15). These functions stem, in part, from the flexible structure of this intrinsically disordered protein, which facilitates its binding to a wide array of ligands (15). This ligand-binding promiscuity suggests that SAA on lipoprotein particles acts as a dynamic protein hub in inflammation control (5, 17). To unravel the molecular basis for SAA functions, the current study explores how binding to various lipids modulates SAA conformation and its interactions with other selected ligands. Unlike most prior studies that explored lipid-free or HDL-bound SAA (reviewed in (6)), our focus is on non-HDL SAA–lipid complexes.

Although most circulating SAA binds reversibly to plasma HDL in inflammation or to LDL and VLDL in diabetes or obesity (11, 18), this does not explain the rapid and dramatic upregulation of SAA in acute phase response, suggesting that other forms of SAA not bound to plasma lipoproteins play a role. SAA can be transiently released from lipoproteins in a lipid-poor/free form (11) that is thought to either be rapidly degraded, bind lipids, or form amyloid (15, 19). Besides HDL-bound and HDL-free SAA, transient non-HDL SAA-lipid nanoparticles can form through phospholipid and cholesterol efflux to SAA from intact cells, either via the ABC transporters ((6, 20, 21, 22) and refs therein) or through detergent-like lipid extraction (23). Moreover, mouse model studies of cutaneous infections showed that SAA solubilized bacterial lipids to form SAA:lipid nanoparticles locally at the injured sites (24).

The latter finding supports our hypothesis that SAA acts as a lipid scavenger that sequesters unprotected lipids at the injured sites. By integrating biophysical studies with the X-ray crystal structures of SAA (16, 25), we postulated that SAA oligomers sequester lipids in a variable hydrophobic cavity (26, 27). Hydrogen deuterium exchange combined with other biophysical studies showed that SAA binds POPC via a concave hydrophobic face formed by amphipathic α-helices h1-h3 (27); this face also binds other lipidic ligands including HDL [(17) and retinol (16). Importantly, SAA-lipid nanoparticles form curved substrates for and remove toxic products of secretory phospholipase A_2_ (sPLA_2_), an acute-phase response protein upregulated concomitantly with SAA (28). This and earlier biochemical studies (29) compelled us to propose that SAA and sPLA_2_ act in synergy to clear unprotected lipids from the sites of injury (30).

Whether this synergy influences amyloid formation by SAA is unknown. However, SAA readily formed amyloid in complexes with oleic acid (OA), which exemplifies free fatty acid generated by sPLA_2_ (30). Amyloid was also formed by SAA fragments in complexes with acidic lyso-phospholipids (31) and by full-size SAA at lysosomal pH in the presence of POPC vesicles (32). Furthermore, lipid-free SAA readily formed amyloid under conditions, where SAA bound to plasma or model HDL was protected from misfolding (32, 33). Conversely, SAA release from HDL upon SAA binding to HS was proamyloidogenic (13). Similar to SAA, release from lipoproteins of other apolipoproteins promoted their misfolding into amyloid (19). Clearly, binding to some but not all lipids protects SAA from misfolding; still, little is known about the effects of specific lipids on the structure, function, and misfolding of SAA.

The current study probes if SAA can solubilize diverse lipids, including mammalian biomembranes, and determines the structural and functional properties of the resulting complexes. We explore how these complexes interact with: *i*) HS mimetics, heparin, and eprodisate; *ii*) CD36, a dual-action scavenger receptor that mediates cellular uptake of lipoproteins (34), binds SAA, and mediates SAA-induced signaling (35). Finally, we determine how lipolysis of these SAA–lipid complexes influences amyloid formation by SAA. The results shed light on the links between the beneficial and the pathologic actions of this enigmatic protein.

## Materials and methods

The current study explored murine SAA 1 (mSAA1) isoform and its human counterpart, hSAA1. This major inducible isoform binds to HDL and forms a protein precursor of amyloid. All methods of protein, lipid, and lipoprotein preparation and analysis are reported in the online Supplement.

## Results

### Structure and stability of SAA complexes formed by solubilization of model lipids

**Fig. 1:**
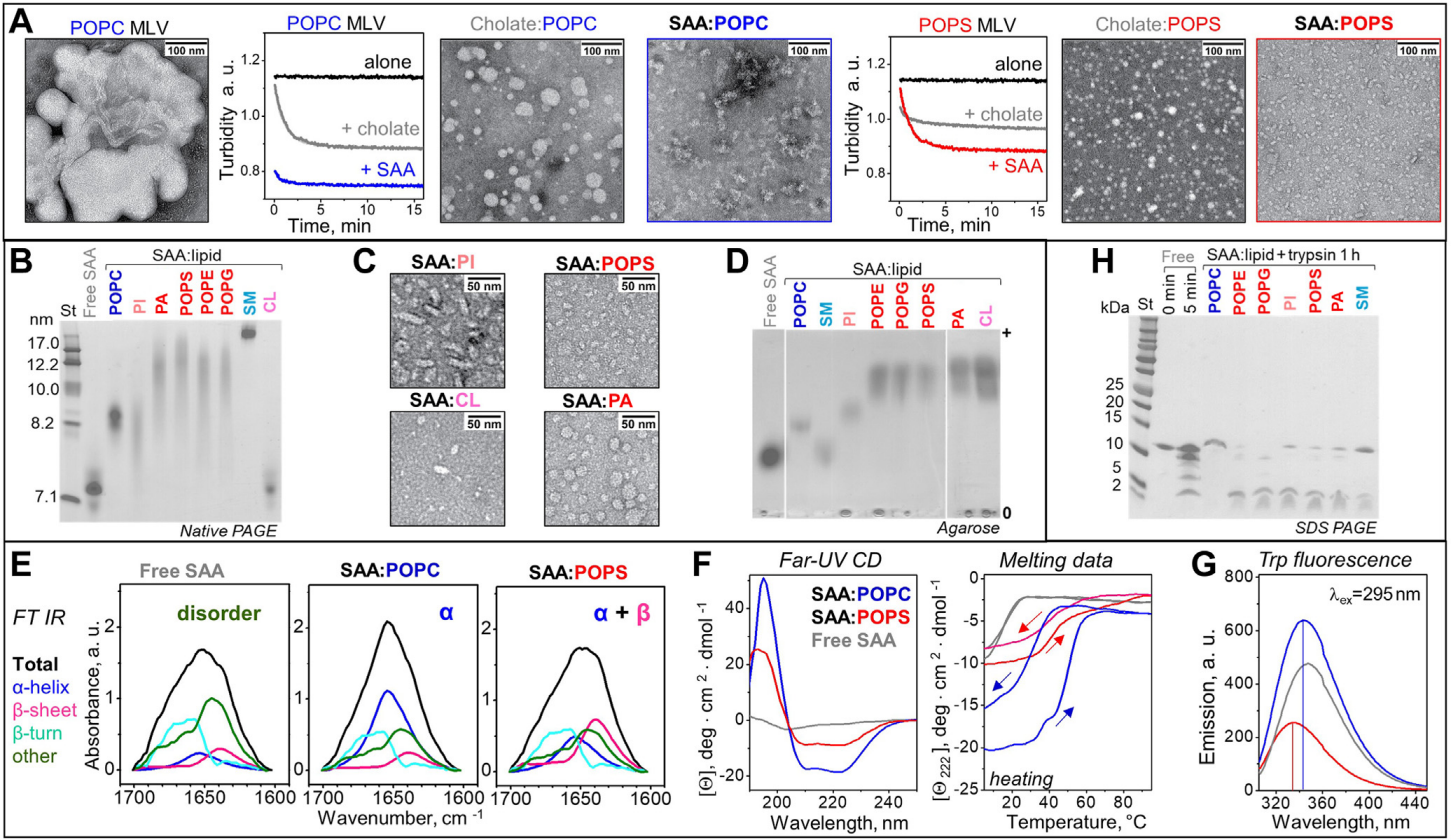
Structure and stability of SAA:lipid complexes formed by solubilization of model lipids. A: MLV clearance for POPC (blue) or POPS (red) during incubation at pH 7.4, 37°C with either mSAA or cholate, monitored by turbidity. Electron micrographs of negatively stained POPC MLVs and of samples after 12 h incubation. B: Native PAGE of SAA:lipid complexes. C: EM of selected complexes; for additional EM data see (30). D: Agarose gel; spliced images are separated by white lines. E: FT IR spectra (black lines) of representative complexes at 22°C; colored lines show individual secondary structural components obtained by spectral deconvolution. F: Far-UV CD spectra at 37°C and the heating/cooling CD data of representative complexes; arrows indicate the directions of temperature changes. Additional FT IR and CD data are shown in supplemental Fig. S1. CD spectra at 25°C and 37°C closely superimposed. G: Tryptophan emission spectra at 37°C of selected complexes. H: SDS-PAGE of SAA:lipid complexes after 1 h of tryptic digestion; free SAA was either intact (0 min) or incubated with trypsin for 5 min. Lipid color coding: POPC (blue); SM (teal), PI (light red); POPS, POPE, POPG, PA (red), CL (pink); and lipid-free SAA (light gray). CL, cardiolipin; EM, electron microscopy; FT IR, Fourier-transform infrared, MLV, multilamellar vesicle; mSAA, murine SAA; PA, phosphatidic acid; PI, phosphatidylinositol; POPE, palmitoyl oleoyl phosphatidylethanolamine; POPG, palmitoyl oleoyl phosphatidylglycerol; POPS, palmitoyl oleoyl phosphatidylserine; SAA, serum amyloid A.

To probe the detergent-like property of SAA, we compared its action on model lipids with that of sodium cholate, a natural detergent from bile. POPC or palmitoyl oleoyl phosphatidylserine (POPS) multilamellar vesicles (MLVs) (∼0.5 μm in size) were incubated with either SAA or cholate at 37°C, pH 7.4. Both SAA and cholate solubilized MLVs within minutes, as indicated by decreased turbidity, and formed 10–40 nm particles seen by electron microscopy (EM); SAA formed smaller particles than cholate (Fig. 1A). The products of lipid solubilization by SAA are henceforth termed as SAA: lipid complexes. Next, we probed the detergent-like action of SAA using MLVs of other phospholipids (supplemental Table S1). At 37°C in detergent-free buffer at pH 7.4, SAA solubilized MLVs of all phospholipids explored, including zwitterionic (POPC, SM) and anionic lipids [POPS, palmitoyl oleoyl phosphatidylethanolamine (POPE), palmitoyl oleoyl phosphatidylglycerol (POPG), phosphatidic acid, cardiolipin (CL), phosphatidylinositol (PI)], to form SAA:lipid complexes. For further studies, such complexes were isolated in the density range 1.06–1.21 g/ml. supplemental Table S2 reports the stoichiometry of these SAA:lipid complexes; native PAGE and/or EM showed heterogeneous particles ranging in size from ∼7.5 nm for SAA:CL to ∼20 nm for SAA:SM (Fig. 1B, C) (30). Agarose gels showed that, as expected, the negative charge on SAA:lipid complexes increased from zwitterionic to anionic lipids, with SAA:PI showing an intermediate charge (Fig. 1D).

Protein conformation in isolated SAA:lipid complexes was explored by Fourier-transform infrared (FT IR), far-UV CD, and fluorescence spectroscopy (Fig. 1E–G; supplemental Fig. S1; supplemental Tables S3–S5). Unlike lipid-free mSAA, which is disordered above 22°C, in complexes with zwitterionic lipids SAA showed increased α-helical content; unexpectedly, SAA in complexes with anionic lipids showed substantial β-sheet content approaching ∼30% (Fig. 1E, F; supplemental Fig. S1; supplemental Table S3). Furthermore, compared to free SAA, for all SAA:lipid complexes tryptophan emission showed a blue shift and increased fluorescence anisotropy (Fig. 1G; supplemental Table S4), indicating decreased polarity of the environment and decreased fluidity, which is consistent with lipid binding. Emission intensity and the mean fluorescence lifetime were much lower for SAA complexes with anionic versus zwitterionic lipids or versus free SAA (Fig. 1G; supplemental Table S5), indicating major differences in the environment of the mSAA tryptophans (W18, W29, W53) upon complex formation with anionic versus zwitterionic phospholipids.

Thermal and proteolytic stability of SAA:lipid complexes was assessed by far-UV CD and tryptic digestion (Fig. 1F, H; supplemental Fig. S3). CD melting data showed large lipid-dependent changes, including hysteresis in the heating and cooling data (greater for zwitterionic lipids, smaller for anionic lipids, and absent from free SAA) and altered midpoint of thermal unfolding ranging from T_m_ = 17°C in free mSAA to T_m,app_ = 45–50°C for SAA:lipid complexes. Under conditions where free SAA was rapidly degraded by trypsin, SAA:lipid complexes were protected, with strongest proteolytic protection exerted by POPC and weakest protection by anionic lipids, particularly POPE and POPG (Fig. 1H). This finding, together with tryptophan fluorescence data, suggests that SAA is better protected in complexes with zwitterionic versus anionic lipids.

### Structure and stability of SAA complexes formed by solubilization of organ lipid extracts

**Fig. 2:**
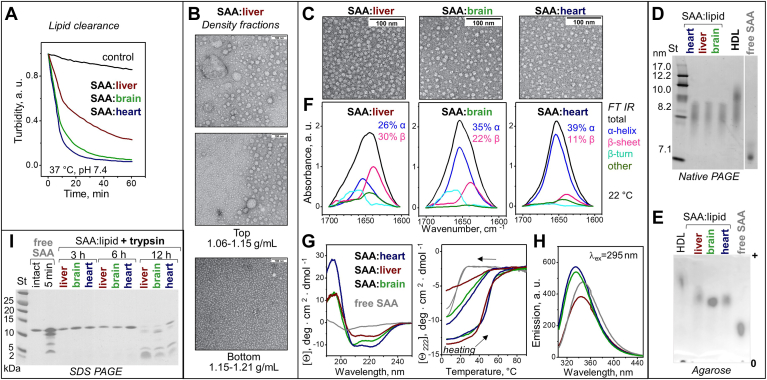
Structure and stability of SAA:lipid complexes formed upon solubilization of organ lipids by mSAA at pH 7.4. A: Clearance of large lipid emulsions by mSAA monitored by turbidity at 37°C; “control” shows data for liver lipid alone. Composition of lipid extracts from liver (wine), brain (green), or heart (blue) is reported in supplemental Fig. S2. B: Wide-field EM images of top (lipid-rich) and bottom (SAA-rich) density fractions formed after 12 h of incubation of SAA with liver lipids. C: The dense fraction containing SAA complexes with various organ lipids was explored using EM. SAA:liver lipid images in panel B (bottom) and C are from the same sample and source image. D: Native PAGE and (E) agarose gel with spliced images separated by a white line. F: FT IR spectra, (G) far-UV CD spectra, heating/cooling data, and (H) tryptophan emission spectra. Selected data for human plasma HDL and for free SAA are shown for comparison. I: SDS-PAGE of SAA:lipid extract complexes after tryptic digestion for 3, 6, or 12 h of (indicated on the lanes); free SAA was either intact or incubated with trypsin for 5 min. EM, electron microscopy; FT IR, Fourier-transform infrared, mSAA, murine SAA; SAA, serum amyloid A.

To determine whether the detergent-like action of SAA extends to biological membranes, we used lipid extracts, which mimic the biomembrane compositions, from bovine liver, bovine heart, and porcine brain (supplemental Fig. S2A). Large lipid emulsions were prepared by vortexing. Differential scanning calorimetry showed a thermotropic phase transition near room temperatures, which for brain lipids extends to physiologic temperatures (supplemental Fig. S2B), and may impact interactions with soluble proteins. At 37°C and pH 7.4, SAA rapidly solubilized the emulsions and formed smaller SAA:lipid particles, as indicated by decreased turbidity (Fig. 2A). To explore these particles, SAA was incubated with emulsions of organ lipids for 12 h as described in Methods, whereupon the samples were fractionated by density in the range 1.06–1.21 g/ml. EM showed that the top fraction (1.06–1.15 g/ml density) contained highly heterogeneous lipid-rich particles (Fig. 2B) that contained little protein. Most protein was recovered in the bottom fraction (1.15–1.21 g/ml) that contained small HDL–size particles and was used for further studies.

For all SAA:lipid extract complexes, EM and native PAGE of this dense fraction showed relatively homogeneous 8–9 nm particles comparable in size to human HDL_3_ (Fig. 2C, D). Supplemental Table S6 shows lipid content in these particles; agarose gel shows that their net negative charge was lower than in human HDL but higher than in free SAA (Fig. 2E). Far-UV CD and FT IR showed that SAA in such complexes with heart lipids was highly α-helical; unexpectedly, the β-sheet content increased at the expense of the α-helix in order SAA:heart < SAA:brain < SAA: liver, reaching ∼30% (Fig. 2F, G; supplemental Table S7). Tryptophan fluorescence revealed that, compared to free SAA, SAA:liver lipid complexes showed decreased emission and a blue shift from 350 to 340 nm, while SAA:heart and SAA:brain lipid complexes showed increased emission and a blue shift to 335 nm (Fig. 2H; supplemental Table S8). SAA:heart and SAA:brain complexes also showed longer mean fluorescence lifetime versus SAA:liver complexes (supplemental Table S9), which probably contributed to the observed differences in the emission intensity. Furthermore, tryptophan anisotropy increased in order: free SAA << SAA:heart < SAA:brain < SAA:liver (supplemental Table S8). Together, these results indicate major structural differences among SAA complexes with lipid extracts from different organs. Despite these differences, all complexes showed comparable thermal and proteolytic stability, which was much greater compared to free SAA (Fig. 2G, I).

In summary, at pH 7.4 and 37°C, SAA can solubilize large (∼0.5 μm) lipid particles of diverse composition, including MLVs of model phospholipids and emulsions of organ lipids, and convert them into lipoprotein-size nanoparticles. Therefore, SAA acts as a protein detergent. Upon lipid solubilization SAA acquires a range of conformations varying in α-helix and β-sheet content (supplemental Tables S3 and S7). Importantly, SAA protection from unfolding and degradation greatly increases upon lipid solubilization. Interestingly, proteolytic protection was greater in SAA complexes with zwitterionic versus anionic lipid, and was much greater in complexes with organ versus model lipids (Figs. 1H and 2I).

### SAA adsorbs onto lipoprotein-size lipid nanoparticles

In inflammation or obesity, SAA binds to plasma HDL, LDL, or VLDL (size range 10–100 nm) and is thought to acquire largely α-helical structure, but the details are obscured by other apolipoproteins. To avoid this interference, we explored SAA interactions with lipid-only nanoparticles in the lipoprotein size range. SAA was incubated at a 1:100 protein:lipid molar ratio either with small unilamellar vesicles (20–30 nm) of POPC or with emulsions (20–60 nm) of POPC:TG or PC:FC (exosome liposomes) for 3 h at 37°C, pH 7.4 (TG, triglyceride; FC, free cholesterol). Although no major SAA-induced changes in the particle size and morphology were detected by EM (supplemental Fig. S3A), all protein adsorbed to the lipid surface, as indicated by size-exclusion chromatography (SEC) and native PAGE (supplemental Fig. S3B, C). Henceforth, these complexes formed by SAA adsorption are termed as SAA+lipid (as opposed to SAA:lipid complexes formed by lipid remodeling and solubilization). Far-UV CD spectra at 25°C showed varied amounts of α-helix and β-sheet in SAA+lipid; for example, SAA+POPC:TG had 58% α-helix and 2% β-sheet, while SAA+POPC:FC had 22% α-helix and 25% β-sheet (supplemental Fig. S3D). This ordered structure unfolded upon heating with T_m,app_ of 40–48°C versus T_m_ = 17°C for free SAA (supplemental Fig. S3E). SDS PAGE following tryptic digestion showed increased proteolytic stability in SAA+POPC:TG and altered proteolytic patterns in other SAA+lipid complexes as compared to free SAA (supplemental Fig. S3F). SAA+POPC:TG had not only higher α-helix content but also higher proteolytic protection compared to other SAA+lipid complexes. Notably, proteolytic protection was greater in SAA:POPC versus SAA+POPC (Fig. 1H vs. supplemental Fig. S3F), consistent with nearly 2-fold higher α-helical content in SAA:POPC versus SAA+POPC observed by CD (blue lines in Fig. 1F and supplemental Fig. S3D).

In summary, SAA adsorbs to the lipid-only nanoparticles of diverse composition, including exosomal liposomes, and folds upon binding. SAA secondary structure and proteolytic stability depend on the lipid composition and on whether the protein has adsorbed to the lipid surface or has extensively remodeled it (e.g., SAA+POPC vs. SAA:POPC).

### SAA binds to micelles of lyso-phospholipids and OA

Increased lipolysis is a hallmark of inflammation and obesity. SAA was proposed to facilitate lipolysis by solubilizing its products, FFA and lyso-lipids (28). To explore SAA interactions with these bioactive lipids, binary complexes were prepared by mixing SAA with micelles of lysophosphatidylcholine, lysophosphatidylethanolamine, lysophosphatidylserine, or OA and were characterized by native PAGE and by CD, FT IR, and fluorescence spectroscopy (supplemental Fig. S4). Intact complexes migrated in the 7.5–9.5 nm size range on the native gel (supplemental Fig. S4A). The protein secondary structure in these complexes was lipid-dependent and was better ordered versus free SAA. Compared to SAA:phospholipid complexes, their lyso-phospholipid counterparts had higher β-sheet and lower α-helix content (Fig. 1E, F; supplemental Fig. S4B, D; supplemental Table S3). Similar trends were observed when comparing SAA complexes with anionic versus zwitterionic phospholipids (Fig. 1E, F; supplemental Fig. S1). Furthermore, tryptophan fluorescence indicated structural differences among SAA complexes with lyso-phospholipids and OA and showed a blue shift and decreased emission intensity versus free SAA (supplemental Fig. S4C).

In summary, at pH 7.4 and 37°C, SAA: *i*) solubilizes large micron-size lipid assemblies with diverse composition transforming them into HDL-size nanoparticles; *ii*) adsorbs to lipoprotein-size lipid particles; and *iii*) binds to micelles of lysolipids and FFA. This versatile lipid-binding ability supports the proposed function of SAA as a lipid scavenger (27, 30). SAA acquires a wide range of conformations in complexes with lipids, from highly α-helical to substantially β-sheet. SAA conformation and proteolytic stability depend upon the lipid composition and the physical nature of the complex (adsorption vs. lipid solubilization). These properties are expected to modulate SAA actions in lipid transport and amyloidogenesis, some of which are described below.

### SAA facilitates binding of intact and oxidized phospholipids to CD36 receptor

In inflammation, binding of SAA-containing HDL (SAA-HDL) to scavenger receptors such as CD36 was proposed to reroute reverse cholesterol transport for cell repair (reviewed in (1)). However, CD36 binding to SAA-only lipoproteins has not been explored. We used SAA complexes with organ lipid extracts, which were either intact or have been oxidized by copper, as CD36 preferentially binds oxidized phospholipids (36). EO6 antibody detected significant levels of endogenous oxidized phospholipids in SAA complexes with liver and brain but not the heart lipids (supplemental Fig. S5A). The rate of copper-induced oxidation of SAA:lipid complexes followed the order: heart < brain < liver (supplemental Fig. S5B). CD36 binding to intact or to copper-oxidized SAA:lipid complexes was explored by ELISA using immobilized extracellular domain of CD36 (supplemental Fig. S5C) as described in Methods. Free SAA and intact SAA complexes with heart lipids showed comparable binding, suggesting that SAA lipidation per se does not significantly alter its affinity for CD36. A small gradual increase in SAA:lipid binding, from heart to brain to liver lipids, correlated with the levels of oxidized phospholipids. Upon oxidation all SAA:lipid complexes showed increased binding. In summary, SAA converts organ lipids into ligands for CD36, whose binding affinity increases upon lipid oxidation. This finding is relevant to inflammation, where both SAA and oxidation levels are elevated, and suggests that cellular uptake via CD36 is not limited to SAA-HDL but extends to other SAA–lipid complexes.

### Heparin binding by SAA:lipid complexes is lipid-dependent

**Fig. 3:**
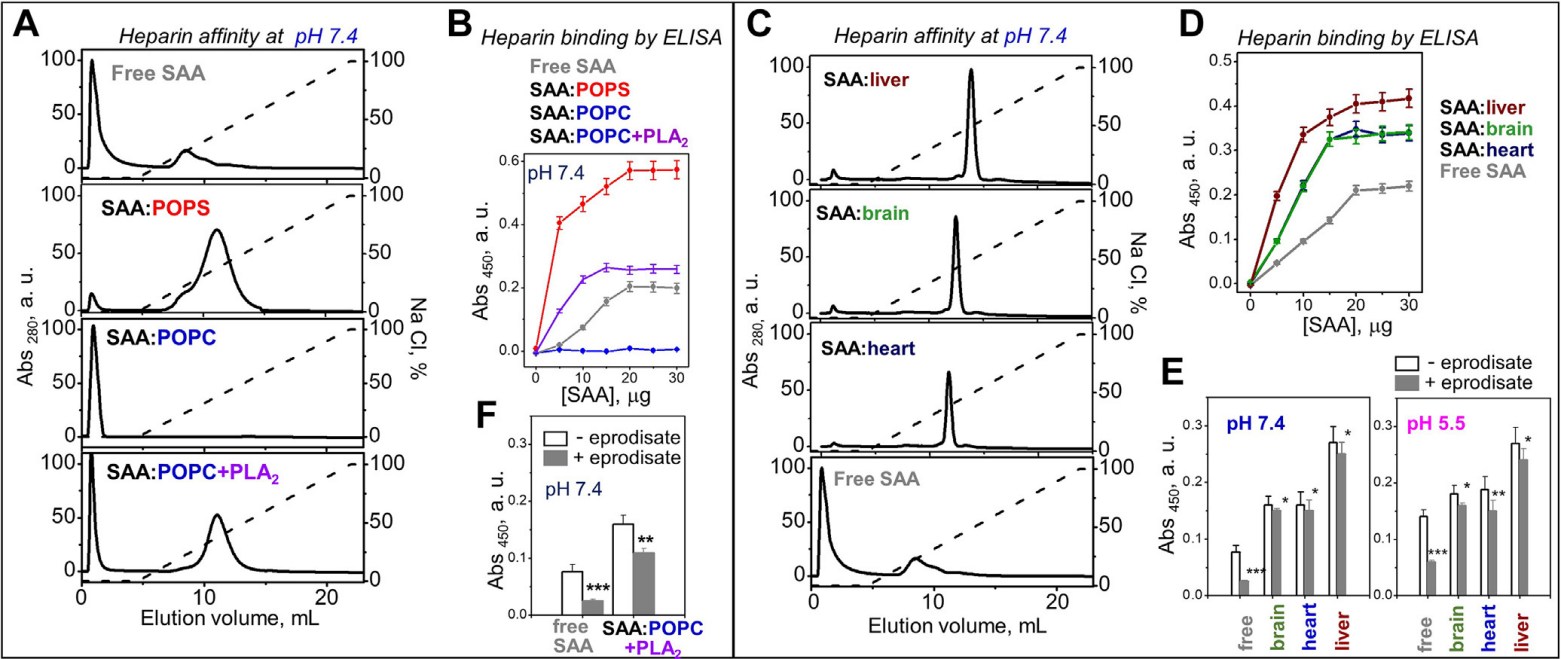
Heparin binding to SAA:lipid complexes. Data for mSAA complexes with model (A and B) or organ lipids (C–E) are shown in comparison with free SAA. In (A and B), SAA:POPS complexes were intact, while SAA:POPC complexes were either intact or had been incubated with sPLA_2_ for 3 h at 37°C, pH 7.4 and reisolated (SAA:POPC+PLA_2_) as described in Methods. A: Heparin affinity profiles; dashed line indicates NaCl concentration (100% stands for 1.01 M NaCl). B: Binding to immobilized heparin monitored by ELISA; similar data for other model lipids are shown in supplemental Fig. S6. C: Heparin affinity profiles, (D) binding to immobilized heparin monitored by ELISA, and (E and F) effects of eprodisate on the binding of SAA:lipid complexes to immobilized heparin monitored by ELISA, with free SAA shown as a control. Panels B, D, E, and F show average values for technical and biological triplicates with associated standard errors. In panels E and F, the values with and without eprodisate are compared; ∗ *P* < 0.05, ∗∗ *P* < 0.01, ∗∗∗ and *P* < 0.001. mSAA, murine SAA; PLA_2_, phospholipase A2; POPS, palmitoyl oleoyl phosphatidylserine; SAA, serum amyloid A.

To explore how SAA:lipid complexes interact with HS, a functional ligand of SAA which is key to AA amyloidosis (1) and to lipoprotein retention in the arterial wall (12, 37), we used heparin, a highly sulfated mimetic of HS. At pH 7.4, heparin affinity chromatography showed that SAA:POPS complexes had higher heparin binding affinity and capacity versus free SAA; in contrast, SAA:POPC showed no binding (Fig. 3A). Solid-phase binding monitored by ELISA confirmed this finding and showed that SAA complexes with anionic phospholipids (POPS, POPG, POPE, phosphatidic acid and, to a lesser extent, PI and CL) bound heparin, while those with zwitterionic phospholipids (POPC and SM) did not bind (Fig. 3B, supplemental Fig. S6). Importantly, heparin affinity chromatography and ELISA showed that SAA and its complexes with organ lipids bound heparin with increasing affinity in order free SAA << SAA:heart ≤ SAA:brain < SAA:liver (Fig. 3C, D), which correlated with increasing levels of oxidized phospholipids (supplemental Fig. S5). This finding suggests a retention mechanism for SAA:lipid complexes in the arterial wall.

Next, we used ELISA with immobilized heparin to probe the effects of eprodisate, a small-molecule mimetic of HS that was proposed to block its interactions with SAA and thereby halt AA amyloidosis (14). At 10 μg SAA (which ensures high sensitivity of the assay) and 1 mM eprodisate, heparin binding by lipid-free SAA was abolished at pH 7.4 and 5.5; however, only a partial decrease was observed for SAA:lipid extract complexes at both pH (Fig. 3E). Therefore, compared to free SAA, in complexes with organ lipids SAA exposes higher affinity heparin-binding sites that cannot be blocked by 1 mM eprodisate, suggesting that a more potent drug is needed to block heparin binding.

### Spontaneous remodeling of SAA:lipid complexes is augmented by acidic pH and heparin

**Fig. 4:**
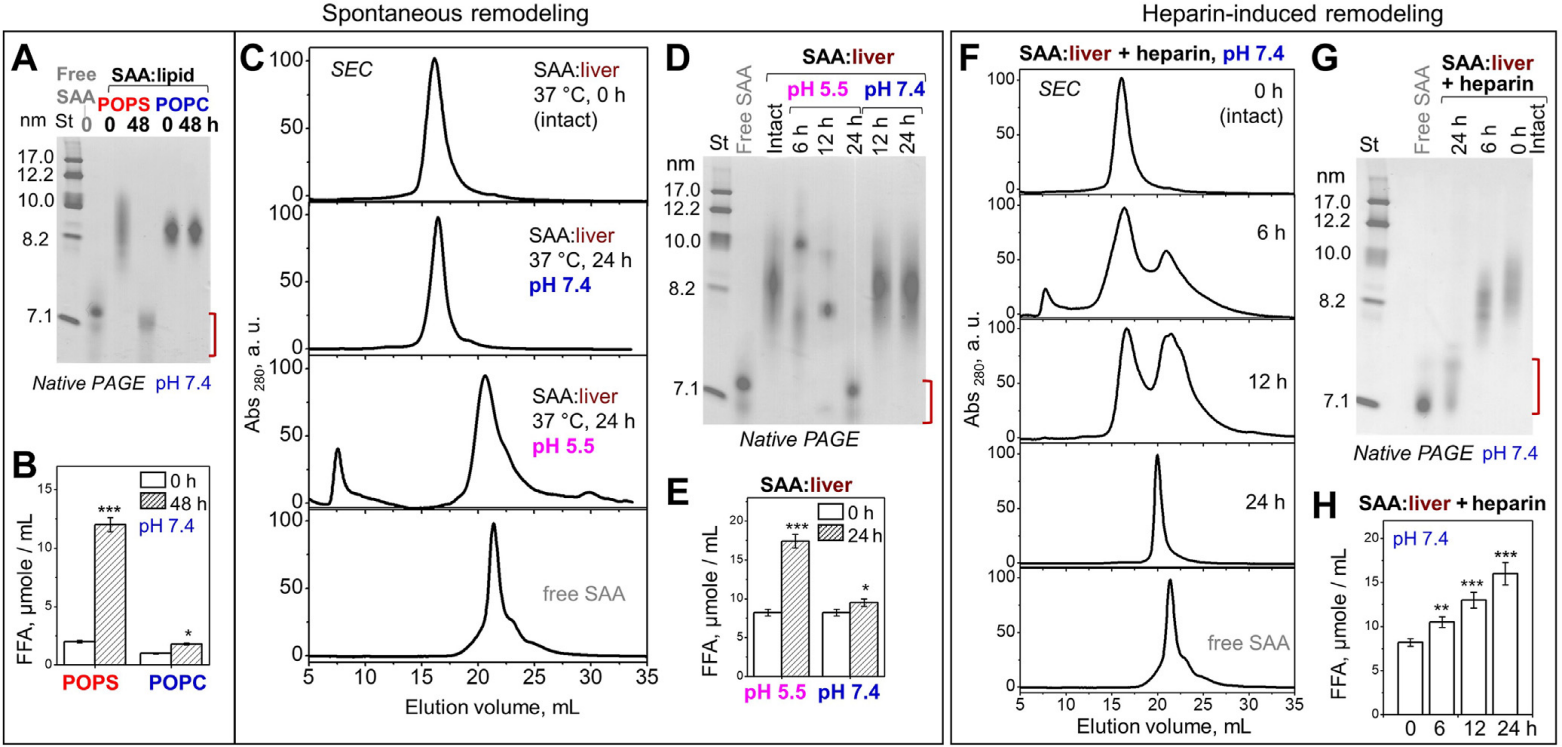
Spontaneous remodeling and lipolysis of SAA:lipid complexes is augmented by heparin binding. Complexes of mSAA with POPC, POPS or liver lipids were incubated at 37°C, pH 7.4 or 5.5 without (A–E) or with heparin (F–H), and total incubation mixtures before (intact, 0 h) or after incubation (for 6, 12, 24, or 48 h as indicated) were assessed; data for free SAA are shown for comparison. A: Native PAGE of SAA:POPC and SAA:POPS; intact lipid-free SAA is shown for comparison. B: FFA content in SAA:POPC and SAA:POPS complexes before and after 48 h incubation compared. C: SEC profiles of SAA:liver lipid complexes that were either intact or had been incubated for 24 h at pH 7.4 or 5.5. D: Native PAGE of SAA–liver complexes, either intact or after 6, 12, or 24 h incubation at pH 7.4 or at pH 5.5. E: FFA content in SAA:liver lipid samples before and after 24 h incubation is compared. F: SEC profiles and (G) native PAGE of SAA+liver lipid complexes intact or incubated for 6–24 h at pH 7.4, 37°C with heparin. H: FFA content in these samples before and after incubation is compared. Values in (B and E) are averages of biological and technical triplicates with associated SEM. Red bracket in (A, D, and G) indicates migration range of lipid-poor SAA. ∗ *P* < 0.05, ∗∗ *P* < 0.01, ∗∗∗ *P* < 0.001. mSAA, murine SAA; POPS, palmitoyl oleoyl phosphatidylserine; SAA, serum amyloid A; SEC, size-exclusion chromatography.

Continuous biochemical changes during lipoprotein metabolism can shift the balance between the lipoprotein core and surface, causing particle fusion, fission, and release of lipid-poor apolipoproteins (38) that are proposed to form protein precursors of amyloid (19). Structural remodeling of plasma lipoproteins may result from multiple factors, including oxidation and hydrolysis at near-physiologic solvent conditions and temperatures (reviewed in (15)). The remodeling of SAA:lipid complexes has not been explored. To probe for spontaneous remodeling, we explored the structural and biochemical integrity of SAA complexes with zwitterionic (POPC), anionic (POPS), or organ (liver) lipids incubated for 24–48 h at 37°C (Fig. 4). Native PAGE, SEC, and FFA assays of the total incubation mixture showed that spontaneous particle remodeling and lipolysis occurred in a lipid- and pH-dependent manner. For a lipid-dependent example, after 48 h incubation at pH 7.4, SAA:POPC complexes remained intact and the FFA content increased but remained relatively low. In contrast, SAA:POPS complexes were remodeled into smaller lipid-poor particles seen on the native PAGE (which migrate close to but are distinct from free SAA, as discussed below), and the FFA content increased 5-fold (Fig. 4A, B). An example of pH-dependent remodeling is SAA:liver lipid complexes, which remained intact after 24 h incubation at pH 7.4; however, at pH 5.5 they were progressively remodeled into larger and smaller particles, including the ∼7 nm lipid-poor species seen at 24 h (Fig. 4C, D). Again, this structural remodeling was accompanied by a significant increase in FFA observed at pH 5.5 but not pH 7.4 (Fig. 4E). Furthermore, spontaneous remodeling may have been accelerated by the oxidized phospholipids in SAA:liver complexes (supplemental Fig. S5A). Together, these findings suggest that spontaneous structural remodeling of various SAA:lipid complexes with a release of lipid-poor SAA is linked to lipolysis, which is accelerated at acidic pH, in complexes with anionic versus zwitterionic lipids and perhaps for oxidized phospholipids.

Binding to heparin via the protein moiety was previously proposed to release SAA from HDL (13) and induce irreversible structural remodeling in other lipoproteins (39). To test if heparin binding promotes the remodeling of SAA:lipid complexes, SAA complexes with liver lipids were incubated with heparin in solution at 37°C for up to 24 h. SEC and native PAGE revealed gradual remodeling of intact 8–10 nm particles into larger and smaller particles including lipid-poor SAA, accompanied by progressive lipolysis (Fig. 4F–H). This finding further supports the link between lipolysis and remodeling of SAA:lipid complexes with release of lipid-poor SAA and shows that heparin binding augments these processes.

In summary, our results reveal a link between lipolysis of SAA:lipid complexes and their structural remodeling with a release of small lipid-poor SAA. We show that both lipolysis and remodeling are faster in SAA complexes containing anionic versus zwitterionic lipids; both processes are much faster at acidic versus neutral pH; and both are augmented by heparin binding at neutral pH (Fig. 4). These findings are relevant to the metabolism of SAA:lipid complexes that are formed at the inflammation sites where local pH may be as low as 5.5. They are also relevant to the role of HS that can either retain SAA:lipid complexes in the extracellular matrix or release lipid-poor SAA.

### Remodeling of SAA:lipid complexes by sPLA_2_

**Fig. 5:**
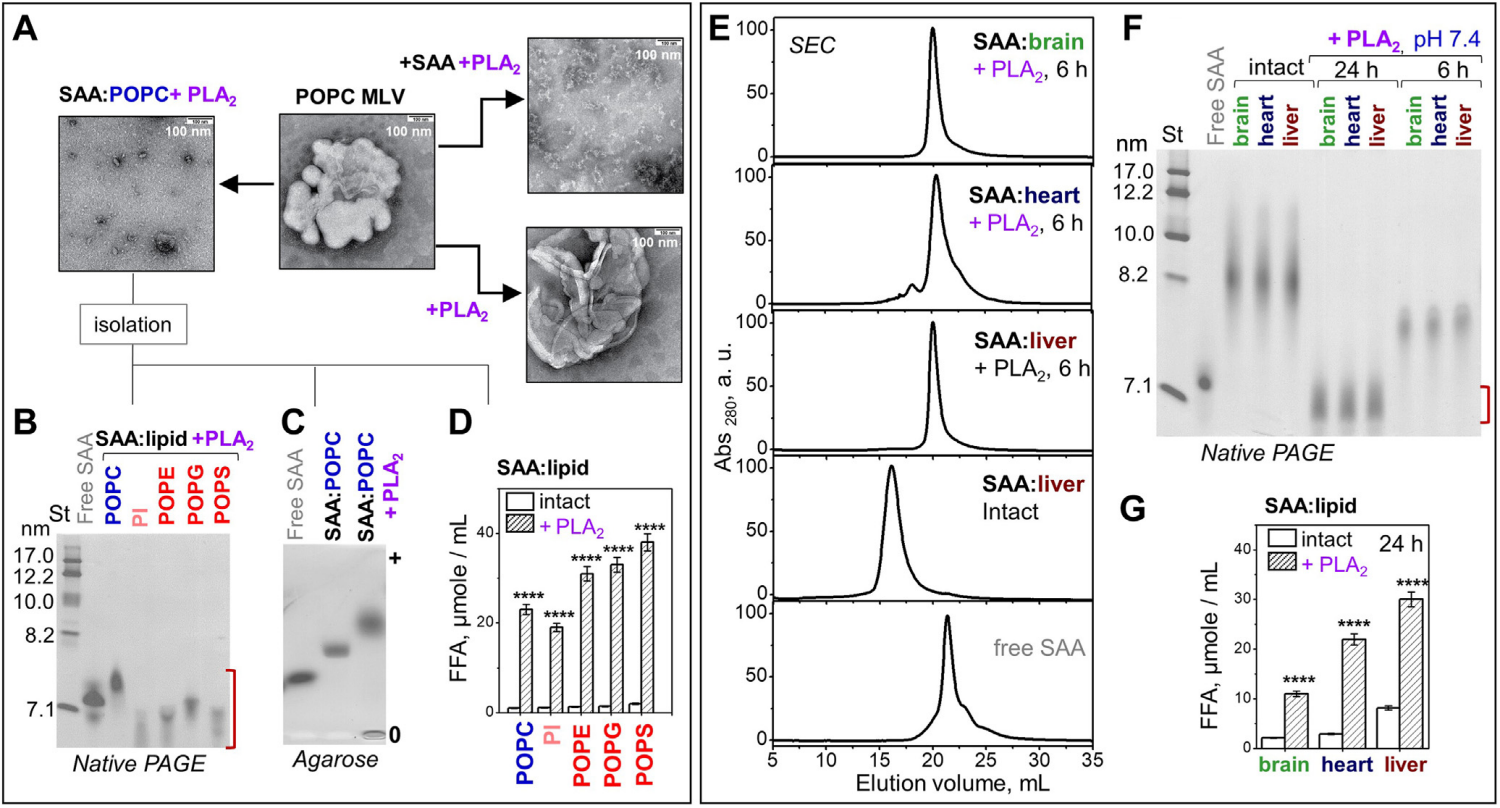
Remodeling of SAA:lipid complexes by sPLA_2_. Complexes of mSAA with model (A–D) or organ lipids (E–G), which were formed by solubilization, were incubated with sPLA_2_ (SAA:lipid +PLA_2_) at 37°C, pH 7.4 and reisolated. Data for lipid-free SAA are shown for comparison. A: EM of POPC MLV alone or after incubation with sPLA_2_ (+PLA_2_), with SAA and sPLA_2_ (+SAA+PLA_2_), or first with SAA to form SAA:POPC and then with sPLA_2_ (SAA:POPC+PLA_2_). POPC MLV data in Fig. 1A and 5A are from the same sample and source image. B: Native PAGE of SAA complexes with various model lipids after 3 h incubation with sPLA_2_. C: Agarose gel of SAA:POPC before and after 3 h incubation with sPLA_2_. D: FFA content in SAA:lipid complexes before (intact) or after incubation with sPLA_2_ is compared. E: SEC of SAA complexes with organ lipids before (intact) or after (+PLA_2_) 6 h incubation with sPLA_2_. F: Native PAGE of SAA complexes with organ lipids before (intact) or after incubation with sPLA_2_ for 6 h or 24 h; red bracket indicates migration range of FFA-rich SAA. G: FFA content in SAA complexes with organ lipids before (intact) or after (+PLA_2_) 24 h incubation with sPLA_2_. ∗ *P* < 0.05, ∗∗ *P* < 0.01, ∗∗∗ *P* < 0.001, and ∗∗∗∗ *P* < 0.0001. EM, electron microscopy; MLV, multilamellar vesicle; SAA, serum amyloid A; SEC, size-exclusion chromatography; PLA_2_, phospholipase A2.

Lipid clearance from the sites of injury was proposed to involve synergistic action of sPLA_2_ and SAA: SAA can solubilize unprotected lipids to generate highly curved substrates for sPLA_2_ and remove its products (30). To explore this synergy, POPC MLVs were incubated at 37°C, pH 7.4 either with sPLA_2_ alone (+PLA_2_) or with both SAA and sPLA_2_ (+SAA+PLA_2_, Fig. 5A). After 3 h the reaction was stopped and the samples were visualized by EM. As expected, sPLA_2_ alone did not remodel MLVs; in stark contrast, a major remodeling into small particles was observed in the presence of SAA. A similar remodeling was observed when SAA was first incubated with POPC MLVs to form SAA:POPC complexes, which were then hydrolyzed for 3 h by sPLA_2_ (SAA:POPC+PLA_2_). To explore the reaction products in detail, SAA:lipid complexes with various model or organ lipids were incubated with sPLA_2_ at pH 7.4, 37°C for 3 h whereupon the reaction was stopped and the lipoproteins were reisolated by density at 1.21 mg/ml for further studies. SAA complexes with POPC, PI, POPE, POPG, and POPS (which are all substrates for sPLA_2_) were hydrolyzed by sPLA_2_, which converted the parent particles (8–18 nm, Fig. 1B) into much smaller particles (6.5–7.5 nm, Fig. 5B) observed by native PAGE. As expected, compared to their parent particles, these small sPLA_2_ products showed a dramatic increase in the net negative charge and in FFA content (Fig. 5C, D).

Similar to model lipids, SAA complexes with organ lipids were progressively remodeled into smaller particles by sPLA_2_ (Fig. 5E, F). FFA content in these particles greatly increased upon remodeling in order brain < heart < liver lipid (Fig. 5G). Native PAGE and SEC showed that, despite different lipid compositions, SAA complexes with various organ lipids were remodeled at similar rates into particles of similar sizes (Fig. 5E, F).

In the next series of experiments we determined the structural and functional properties of small lipid-poor SAA species generated from SAA:lipid complexes upon sPLA_2_ hydrolysis. To this end, SAA:POPC complexes at pH 7.4, which were hydrolyzed by sPLA_2_ and re-isolated (SAA:POPC+PLA_2_), were explored by FT IR, CD, and fluorescence spectroscopy and by limited proteolysis (supplemental Fig. S7). Compared to their parent SAA:POPC particles, the small SAA:POPC+ PLA_2_ particles showed decreased α-helical content (which was still much higher vs. free SAA) and a slight increase in the β-sheet content; a blue shift and decreased intensity in tryptophan emission; decreased hysteresis in the heating/cooling data; and decreased proteolytic stability (supplemental Fig. S7A–D). Notably, similar trends were observed when comparing SAA complexes with anionic versus zwitterionic lipids (Fig. 1, supplemental Fig. S1), suggesting similar conformational adaptation of SAA in complexes enriched with different negatively charged lipids (anionic phospholipids or FAA). Moreover, similar trends were observed in SAA complexes with lyso-phospholipids versus corresponding phospholipids (supplemental Fig. S4; Fig. 1; supplemental Table S3). These findings support the idea that lipid charge is a major determinant for the SAA conformation in lipid complexes and suggest that both FFA and lyso-lipids contribute to the conformational changes in SAA:lipid complexes observed upon their hydrolysis by sPLA_2_.

Next, we probed if the small SAA:POPC+PLA_2_ particles retained the capacity to bind lipids. The lipid clearance and SEC data demonstrated that, under conditions where free SAA rapidly cleared POPC MLVs or absorbed to the surface of POPC small unilamellar vesicles, SAA:POPC+PLA_2_ showed no lipid solubilization and very little adsorption (supplemental Fig. S7E, F). Therefore, although SAA:POPC+PLA_2_ migrated close to free SAA on the native gel and SEC, they were structurally and functionally distinct.

Next, we explored the heparin-binding affinity of SAA:POPC+PLA_2_ at pH 7.4. Heparin affinity chromatography and ELISA revealed that compared to SAA:POPC (which showed no binding) or free SAA (which showed some binding), SAA:POPC+PLA_2_ bound heparin with increased affinity and capacity (Fig. 3A, B). In the presence of 1 μM eprodisate, heparin binding was only partially decreased for SAA:POPC+PLA_2_ (Fig. 3F). Together, the results show that, despite their increased net negative charge, SAA complexes containing large amounts of either anionic lipids or FFA and lyso-phospholipids bound heparin, while their zwitterionic counterparts did not bind (Fig. 3, supplemental Fig. S6). This suggests that heparin binding reflects protein conformational changes in SAA:lipid complexes induced by the negatively charged lipids, perhaps with a contribution from lyso-lipids.

In summary, the small SAA:POPC+PLA_2_ species generated upon hydrolysis of SAA:POPC complexes by sPLA_2_ have mixed α-helix/β-sheet conformations that are more stable than free SAA but less stable than SAA:POPC; have high-FFA content; do not bind additional lipids; and have increased heparin-binding affinity (Fig. 5B–D, supplemental Fig. S7). Small (≤7 nm) FFA-loaded SAA species are also generated upon sPLA_2_ hydrolysis of SAA complexes with organ lipids (Fig. 5E–G). Whether such species provide protein precursors of amyloid will be discussed later.

### Human SAA:lipid complexes compared to murine SAA:lipid complexes

To test whether the lipid complexes with human SAA have shared properties with their murine counterparts, and thereby test the relevance of this study to human physiology, we explored hSAA:lipid complexes with model and organ lipids. The complexes were generated by solubilization and the results are reported in supplemental Fig. S8. Briefly, at pH 7.4, 37°C, both hSAA and mSAA spontaneously and rapidly (in minutes or faster) solubilized various lipids, including MLVs of all model phospholipids and large emulsions of organ lipid extracts, to form heterogeneous lipoprotein-size nanoparticles (supplemental Fig. S8B, C). The resulting hSAA:lipid complexes, which were explored for heart, brain, and liver lipids, showed shared properties with their mSAA counterparts. These include spontaneous remodeling of SAA:lipid complexes into smaller particles observed at pH 5.5 but not pH 7.4; lipolysis and remodeling of SAA:lipid complexes into smaller particles by sPLA_2_ at pH 7.4; binding to heparin and to CD36, which increased in order free SAA < SAA:heart < SAA:brain < SAA:liver; and the ability of 1 mM eprodisate to fully block heparin binding by lipid-free SAA but only partially inhibit it for SAA:lipid complexes (supplemental Fig. S8A–G). These results suggest that key conclusions from detailed studies of mSAA:lipid complexes can be extrapolated to hSAA.

### Amyloid formation in SAA complexes with model lipids

Lastly, we determined how SAA:lipid complexes influence amyloid formation. Our working hypothesis was that complexes with high-temporal stability protect the protein from misfolding into amyloid; however, these protective properties are lost in complexes that undergo remodeling and release lipid-poor SAA on a time scale typical for amyloid formation. To test this idea, we explored selected SAA:lipid complexes that either showed high-temporal stability or underwent rapid remodeling under amyloid-promoting conditions (in the current study we used 48 h incubation at 37°C with stirring). Lipid-free SAA readily formed amyloid under these conditions and was used as a control. After incubation, the samples were centrifuged and the pellets were resuspended for structural studies. Amyloid formation was monitored using several techniques: *i*) fluorescence of thioflavin T (ThT), whose emission increases upon binding to amyloid-like structure; *ii*) binding of antibodies specific to amyloid oligomers (A11) or fibrils (OC); formation of intermolecular cross-β-sheet with spectral signatures in *iii*) far-UV CD and iv) FT IR; and v) EM of negatively stained samples. Structural integrity of SAA upon incubation was verified by SDS PAGE (supplemental Fig. S9A). The results are reported in supplemental Figs. S6 and S9.

**Fig. 6:**
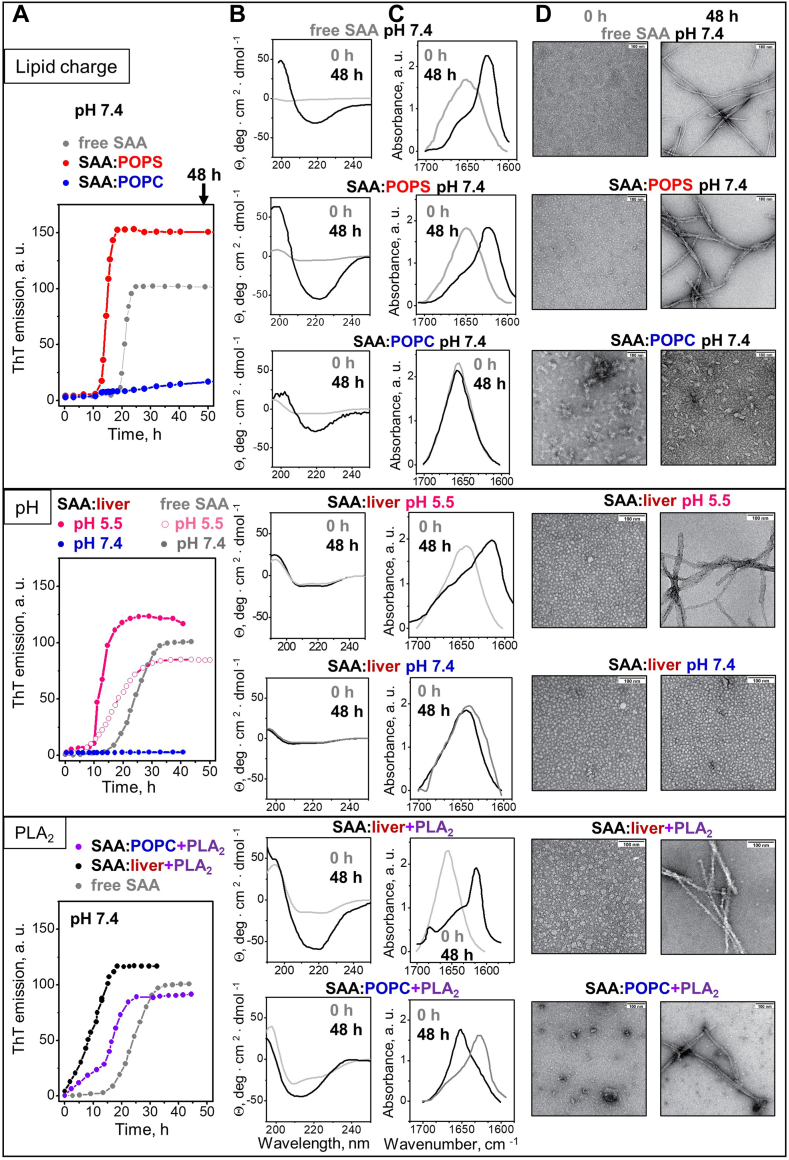
Effects of lipids, pH, and lipolysis on amyloid formation upon incubation of SAA:lipid complexes. Three groups of data, (i) to (iii), are shown top to bottom for the following samples. (i) SAA:POPC (blue), SAA:POPS (red), and free SAA (gray, control) incubated at pH 7.4. (ii) SAA:liver lipids incubated at pH 5.5 (pink) or 7.4 (blue), with data for free SAA at corresponding pH shown as controls. (iii) SAA:POPC that had been treated with sPLA_2_ and reisolated (SAA:POPC+PLA_2_), SAA:liver lipids that had been treated with sPLA_2_ and reisolated (SAA:liver+PLA_2_, black), SAA:POPC that had been treated with sPLA_2_ and reisolated (SAA:POPC+PLA_2_, violet) and free SAA (control, gray). The following data types are shown left to right: A: Time course of amyloid formation during sample incubation under amyloid-promoting conditions (37°C with stirring) monitored by ThT emission. After incubation for 48 h, the sample pellets were collected and used for further studies. B: Far-UV CD and (C) FT IR spectra of samples before (gray, 0 h) and after incubation (black, 48 h). D: EM images of the samples before (0 h) and after incubation (48 h). Scale bar is 100 nm. Additional relevant data are shown in supplemental Fig. S9. Data for SAA:POPC in Fig. 1A and 6D are from the same sample and image source; the same applies to SAA:POPC+PLA_2_ data in Fig. 5A and 6D and to SAA:liver data in Fig. 2B and 6D. EM, electron microscopy; FT IR, Fourier-transform infrared; POPS, palmitoyl oleoyl phosphatidylserine; SAA, serum amyloid A; sPLA2, secretory phospholipase A2; ThT, thioflavin T.

First, we incubated at pH 7.4 SAA:POPC, which remained stable, and SAA:POPS, which showed spontaneous remodeling with release of lipid-poor SAA (Fig. 4). Like free SAA, SAA:POPS showed a sigmoidal increase in ThT fluorescence, suggesting nucleation-growth reaction characteristic of amyloidogenesis (Fig. 6A). The incubation products of SAA:POPS bound OC (supplemental Fig. S9C) showed large spectral changes in far-UV CD and FT IR characteristic of intermolecular cross-β-sheet, and showed amyloid fibrils by EM (Fig. 6D). In stark contrast, SAA:POPC complexes remained structurally invariant and no amyloid was detected after 48 h (Fig. 6). However, prolonged (∼1 month) incubation at pH 7.4, 37°C of either SAA:POPC or SAA+POPC complexes resulted in their remodeling and amyloid formation (supplemental Fig. S9E). These data support our hypothesis that lipid complexes protect SAA from misfolding in amyloid only for as long as these complexes remain stable.

Further support came from ThT measurements taken after 48 h incubation at pH 7.4 of SAA complexes with various model lipids. Complexes with anionic phospholipids, which were remodeled during 48 h, showed a significant increase in ThT emission indicative of amyloid formation; in contrast, complexes with zwitterionic phospholipids, which remained stable during 48 h, showed no significant increase (supplemental Fig. S9B). These results further support amyloid formation only in SAA:lipid samples that undergo remodeling and release of lipid-poor SAA.

Next, we incubated SAA:liver lipid extract complexes either at pH 7.4, where they showed high temporal stability, or at pH 5.5, where they were rapidly remodeled with release of lipid-poor SAA seen in 48 h (Fig. 4). SAA:liver lipid samples showed amyloid formation at pH 5.5 but not at pH 7.4, while free SAA readily formed amyloid in both pH environments (Fig. 6). This finding verifies our hypothesis for organ lipids.

Finally, we incubated at pH 7.4 SAA:POPC and SAA:liver lipid complexes that were either intact or had been hydrolyzed by sPLA_2_ and thereby remodeled with the release of lipid-poor SAA (Fig. 5B, F). Unlike the untreated complexes, which remained intact and did not form amyloid after 48 h incubation, sPLA_2_-treated samples readily formed amyloid (SAA:POPC+PLA_2_ and SAA:liver+PLA_2_, Fig. 6). To determine if particular sPLA_2_ product(s) were responsible for amyloid formation, complexes of SAA with lysophosphatidylcholine, lysophosphatidylethanolamine, lysophosphatidylserine, or OA were incubated at pH 7.4 for 48 h. Antibody binding and EM analysis detected amyloid fibrils in all samples, suggesting that SAA in complexes with both lyso-phospholipids and FFA were conducive to amyloid formation (supplemental Fig. S9F, G).

Taken together, the results support our hypothesis: SAA:lipid complexes protect from amyloid formation as long as they are stable. Conversely, release of lipid-poor SAA species from such complexes upon their remodeling, either spontaneous (as in complexes with anionic lipids or at acidic pH) or enzymatic (as in sPLA_2_-treated complexes) facilitates amyloid formation. Additionally, our results reveal a new aspect of the synergy between SAA and sPLA_2_: by hydrolyzing SAA:lipid complexes, sPLA_2_ releases small metastable FFA-rich SAA species, thus generating the protein precursor of amyloid (Figs. 5 and 6).

## Discussion

This study shows that SAA is a powerful natural detergent that rapidly solubilizes diverse membrane lipids, including mammalian organ lipids, and spontaneously converts them into lipoprotein-size nanoparticles at pH 7.4, 37°C (Figs. 1A and 2A, supplemental Fig. S8A). Similar nanoparticles are expected to transiently form at the sites of injury, infection, and inflammation, where SAA levels are elevated and cellular debris requires rapid removal to permit tissue healing (27, 30). In fact, SAA-lipid nanoparticles formed transiently at the sites of acute infection in mouse model studies (24). Transient non-HDL SAA-lipid nanoparticles were also reported in several cell-based studies (6, 20, 22) and references therein). The current work explores the links between the structure and function of SAA-lipid nanoparticles and amyloid formation. The results show that SAA:lipid nanoparticles formed through lipid solubilization provide substrates for sPLA_2_ (30) (Fig. 5) and sequester its toxic products, lyso-phospholipids and FFA (supplemental Fig. S4) (30, 31), thus facilitating enzymatic lipolysis while protecting from lipotoxicity. SAA:lipid nanoparticles also act as ligands for cell receptors such as CD36 (supplemental Figs. S5 and S8) and heparin (Fig. 3). These findings suggest that the SAA-containing nanoparticles can be taken up by macrophages and other CD36-expressing cells or be retained in the extracellular matrix via HSPGs. Together, the results strongly support the role of SAA as a primordial lipid scavenger that clears unprotected biomembranes by rapidly solubilizing them and directing their lipolysis, safe removal, and cellular uptake. Moreover, SAA can adsorb without remodeling to the surfaces of lipoproteins (6, 32) and references therein) and other lipoprotein-size nanoparticles, such as exosomal liposomes (supplemental Fig. S3), and thereby modulate their metabolism.

The current study reveals that SAA conformation varies widely in different lipid complexes; depending on their lipid composition, SAA:lipid complexes vary not only in their α-helix (from ∼20 to ∼50%) but also in their β-sheet content (from ∼0 to ∼30%, supplemental Tables S3 and S7). This contrasts with the X-ray crystal structures of lipid-free or retinol-bound mSAA or hSAA, which contain ∼75% α-helix and no β-sheet (16, 25). Conversely, the cryo-EM structures of tissue-derived mSAA or hSAA amyloid fibrils contain cross-β-sheet and no α-helix (40, 41). Notably, sequence-based analyses predict amphipathic α-helices and loops but no β-sheets in SAA, and the solution conformation in the presence of the protein folding osmolyte trimethylamine N-oxide is highly α-helical (27). Although prior CD studies of various SAA forms reported a mixed α-helix/random coil conformation with some β-sheet (42, 43), the details were unclear. The current study reveals that SAA shows higher β-sheet and lower α-helix content in less stable complexes with anionic phospholipids or with FFA and lyso-phospholipids versus their zwitterionic counterparts (Figs. 1 and 5; supplemental Figs. S1 and S7). Such a conformational variability is a hallmark of intrinsically disordered proteins, which facilitates ligand binding promiscuity (44). Conformational variability allows SAA to recruit a wide range of ligands for diverse functions (5, 15, 26) including lipid scavenging. Although the exact SAA conformations in lipid complexes are unknown, H-D exchange combined with molecular dynamics simulations indicated that stable α-helices in SAA:POPC complexes are localized to the N-terminal ∼65 residues, while the interhelical linker at residue 32 has the propensity to form a β-hairpin when dissociated from the lipid, potentially initiating protein misfolding in amyloid (27). Since SAA complexes with anionic phospholipids or with FFA and lyso-phospholipids have greater propensity to form amyloid (Fig. 6), substantial β-sheet content in these complexes perhaps including this β-hairpin may facilitate amyloid formation.

In several other amyloidogenic proteins (α-synuclein, Aβ peptide, insulin, etc.), anionic lipids have also been reported to promote β-sheet formation and aggregation, while zwitterionic lipids tend to block it, although the details are protein-specific ((45) and references therein). Unlike SAA, the proteins in those studies interact with lipid vesicles but do not solubilize lipids. A potential basis for this intriguing similarity stems from the ability of anionic lipids to form micelles that can shield the exposed hydrophobic surfaces in amyloids; such micelles have been observed in cryo-EM structures of lipidic α-synuclein fibrils and are expected to occur in other lipidic amyloids (46). A similar mechanism may potentially contribute to amyloid formation in SAA complexes with other micelle-forming lipids, FFA, and lyso-phospholipids (Fig. 6; supplemental Fig. S9).

The lipid-dependent protein conformation observed in SAA–lipid complexes will likely influence the interplay between SAA misfolding, proteolysis, and binding to various functional ligands. In fact, data show that SAA forms stable complexes with organ lipids or model zwitterionic lipids that protect the protein from proteolysis and misfolding (Figs. 1, 2 and 6). Conversely, in complexes containing anionic lipids and/or large amounts of FFA and lyso-phospholipids, SAA adopts less stable conformations, which provide decreased protection from proteolytic degradation and from misfolding in amyloid (Figs. 1 and Fig. 4, Fig. 5, Fig. 6; supplemental Table S3). SAA interactions with its functional ligands such as heparin are also lipid-dependent. Although the binding of SAA:lipid complexes to heparin is mediated by the protein, it depends critically on the lipid composition and is significant only in complexes containing anionic phospholipids or FFA and lyso-phospholipids, but not zwitterionic lipids alone (Fig. 3, supplemental Fig. S6). Therefore, both lipid composition and protein conformation influence functional interactions of SAA:lipid nanoparticles and their metabolic fate.

Notably, SAA conformation and proteolytic stability in lipid complexes depend not only on the lipid composition but also on the binding mechanism, that is adsorption to the existing lipid nanoparticles (SAA+lipid, which is arguably a proxy for SAA bound to plasma lipoproteins) versus de novo nanoparticle formation (SAA:lipid, which forms through detergent-like action). Compared to SAA+lipid, SAA:lipid have higher α-helical content and higher proteolytic stability (compare SAA:POPC in Fig. 1 with SAA+POPC in supplemental Fig. S3). This suggests that, like other apolipoproteins (38), SAA on the lipoprotein surface is kinetically trapped, an assumption that is supported strongly by the hysteresis in the melting data of SAA complexes with organ lipids (Fig. 2G). Together, the results suggest that the mechanism of the complex formation (adsorption vs. solubilization) affects the SAA conformation and thereby may modulate the metabolism of the SAA–lipid complexes.

The current study reveals a previously unknown role of sPLA_2_ in SAA amyloidogenesis. While stable SAA:lipid complexes protect the protein from misfolding, lipolysis of these complexes augments heparin binding and facilitates structural remodeling and amyloid formation at physiologic pH (Figs. 5 and 6). Specifically, sPLA_2_ lipolysis of SAA:lipid complexes releases small SAA species that are rich in FFA and lyso-phospholipids and have the following: *i*) higher β-sheet and lower α-helix content than the parent SAA:lipid complexes (supplemental Fig. S7A, B); *ii*) increased heparin binding affinity than either free SAA or the parent complexes; and *iii*) proteolytic stability that is intermediate between that of free SAA (which is rapidly degraded, as seen after 5 min with trypsin, Fig. 1H) and the parent complexes (which are highly protected, as seen after 1 h with trypsin, supplemental Fig. S7D). We posit that such an intermediate stability facilitates accumulation of these misfolded species (vs. their rapid degradation), which is prerequisite for amyloid formation. Notably, these small metastable SAA species are unable to bind additional lipids and convert into larger more stable complexes (supplemental Fig. S7E, F); this contrasts with free SAA that is either rapidly degraded or binds lipids to form stable complexes. Finally, these metastable species readily form amyloid (Fig. 6, bottom panels). We conclude that the synergy between SAA and sPLA_2_ extends from their beneficial function in removing unprotected biomembranes, to the generation of metastable misfolded SAA species with high propensity to accumulate and form amyloid.

Heparin plays a multifaceted role in this pathogenic process by releasing SAA from the lipoprotein surface (13) and acting as a template for amyloid formation (47). Current data indicate that the small metastable SAA species generated by sPLA_2_ have increased affinity for heparin binding, posing a challenge for blocking this binding with a small-molecule drug eprodisate (Fig. 3A, F). Additionally, the lipid-dependent binding of SAA complexes to HS may promote their proatherogenic retention in the extracellular matrix of the arterial wall, which may contribute to the link between inflammation and atherosclerosis.

## Data availability

The datasets generated and analyzed during the current study (gels, spectra, chromatography traces, electron micrographs etc.) are not publicly available due to lack of databases specializing in these types of data but are available from the authors upon reasonable request.

## Supplemental data

This article contains supplemental data (48, 49, 50, 51, 52, 53, 54, 55, 56).

## Conflict of interest

The authors declare that they have no conflicts of interest with the contents of this article.
