# Supplementary material for: Lipid clearance and amyloid formation by serum amyloid A: exploring the links between beneficial and pathologic actions of an enigmatic protein
Source: J Lipid Res. 2023 Aug 19;64(9):100429. doi: 10.1016/j.jlr.2023.100429 (PMC10509712; doi:10.1016/j.jlr.2023.100429)
Supplement: Supplemental Data [file mmc1.pdf]

SUPPLEMENT

Lipid Clearance and Amyloid Formation by Serum Amyloid A: Exploring the links between beneficial and pathologic actions of an enigmatic protein

Shobini Jayaraman, Angela Urdaneta, Esther Bullitt, Marcus Fändrich, Olga Gursky

CONTENT

- **Methods**
- **Supplemental Tables 1 – 9**
- **Supplemental Figures 1 – 9**
- **Supplemental References**
- **Resources Tables**

METHODS

Proteins Recombinant full-length murine SAA1 (mSAA), a major amyloidogenic isoform that binds HDL, was used throughout this study; mSAA was expressed in *E. coli* and purified as previously described (1). Recombinant human SAA1 (hSAA), which contained an extra N-terminal Met compared to plasma protein, was obtained commercially (Preprotech, #300-53). Compared to mSAA1, hSAA1 contained an additional N-terminal residue, R1; hSAA1 and mSAA1 had 73% amino acid sequence identity and 83% similarity. For hSAA, protein purity was ≥ 98% by SDS PAGE and HPLC analyses according to the manufacturer. Protein amino acid sequences are as follows:

mSAA1: GFFSFIGEAFQGAGDMWRAYTDMKEAGWKDGDKYFHARGNYDAAQRGPGG

VWAAEKISDARESFQEFFGRGHEDTMADQEANRHGRSGKDPNYYRPPGLPAKY

hSAA1: MRSFFSFLGEAFDGARDMWRAYSDMREANYIGSDKYFHARGNYDAAKRGPGG

VWAAEAISDARENIQRFF GHGAEDSLAD QAANEWGRSG KDPNHFRPAGLPEKY

Stock solutions of mSAA or hSAA (5-10 mg/mL protein in double-distilled (d.d.) water) were dialyzed extensively against d.d. water to remove residual solvent. Prior to experiments, protein stock solutions were diluted in 10 mM sodium phosphate buffer, pH 7.4 (which was the standard buffer used throughout this study unless otherwise stated) and were centrifuged at 10,000 r.p.m. for 10 min using Beckman Coulter Microfuge 18 to remove insoluble aggregates.

Resources Tables at the end of this Supplement list the antibodies, assays, lipids, enzymes, and other resources used in this study.

Preparation of SAA:lipid complexes Lipids were solubilized in chloroform:methanol (2:1 v/v) and dried under N_2 stream, followed by overnight drying under vacuum at 4 °C to form a thin film. The film was re-suspended in 50 mM PBS, pH 7.4 with vigorous vortexing multiple times to ensure a uniform suspension of MLVs (for anionic or zwitterionic phospholipids) or emulsions (for organ lipid extracts). For spontaneous lipid clearance by SAA, aliquots of this stock suspension were used. To prepare SUVs, the suspension was sonicated on ice using Branson sonicator for 45-60 min until it became clear. To prepare small emulsion particles of exosomal liposomes, lipids were sonicated on ice for 3-5 min to ensure complete mixing (2). To prepare small emulsions of TG-PC, mixtures of triolein with egg yolk PC (4.5:1, wt:wt) were sonicated in PBS and particles were isolated by density gradient centrifugation at 1.006-1.065 g/mL NaCl (3). Lipid composition in purified emulsions was TG:PC 70:30, wt/wt, as quantified using a Bioassay system with Enzychrom phospholipid and TG assay kits, EPLP-100 and ETGA-200.

Lipid suspensions prepared by these methods were incubated with SAA at 37 °C for 12 h. SAA: lipid ratio was 1:100 mol/mol for model lipids and SUVs, and 1:4 wt/wt for organ lipid extracts.

For lyso-phospholipids or OA, SAA (0.5 mg/mL) was incubated with freshly prepared micelles of lyso-lipids or sodium oleate (1 mmol) in standard buffer for 2 h at 37 °C. Lyso-phospholipid solution in chloroform was dried under N_2 and the film was re-suspended in 10 mM PBS, pH 7.4 by vortexing. OA stock solution (1 M) was freshly prepared by mixing in 10 mM PBS, pH 7.4. The micelles were used immediately for further studies.

Delipidation To determine lipid composition, lipid dry weight was quantified by extraction following published protocols (4). Briefly, 1 mL of 0.5 mg/mL samples were mixed with 5 mL of 8:4:3 parts of chloroform : methanol : 0.9% NaCl. The mixtures were centrifuged at 2000 *g* for 10 min. The organic phase was evaporated under N_2 gas. The dried lipid was weighed using analytical balance (Mettler Toledo XSR105).

Lipid clearance kinetics For model phospholipids, the time course of MLV clearance by mSAA at 37 °C was monitored by turbidity at 325 nm using a Varian Cary-3500 UV-vis spectrophotometer. SAA (20 µg/mL) was rapidly mixed with phospholipid MLVs (80 µg/mL lipid) in standard buffer, and turbidity was recorded in real time as large MLVs were converted into smaller lipoprotein nanoparticles. MLVs alone were used as negative controls; as positive controls clearance experiments were performed using 80 µg/mL of freshly prepared sodium cholate. For large emulsions of organ lipid extracts, clearance experiments using mSAA or hSAA were performed under identical conditions; the results are reported in Fig. 2a and supplementary Fig. 8a.

Calorimetry Thermotropic phase transitions in lipid acyl chains were assessed by differential scanning calorimetry. Briefly, lipid emulsions (1 mg/mL organ lipid extract in 50 mM PBS, pH 7.4) were heated from 5 °C to 90 °C at a rate of 90 °C/h, and the heat capacity $C_p(T)$ was recorded using VP-DSC microcalorimeter (MicroCal, MA, USA). The buffer baselines were subtracted from the data. ORIGIN software was used for the data collection and display.

Limited proteolysis To test for proteolytic stability, SAA, which was either lipid-free or in complexes with lipids, was incubated with trypsin at 1:200 mg:mg substrate:enzyme ratio in standard buffer at pH 7.4, 37 °C. Tryptic digestion was stopped by adding 2 mM of phenylmethylsulfonyl fluoride, a serine protease inhibitor, after either 5 min (for lipid-free SAA, which was rapidly digested) or 1 h (for lipid-bound SAA, which was digested much more slowly). The extent of proteolysis was assessed using SDS PAGE.

Gel electrophoresis For non-denaturing PAGE, Novex™ 4-20% Tris-glycine gels (Invitrogen) were loaded with 6 μg protein per lane and run to termination at 1,500 V·h under non-denaturing conditions in Tris-glycine buffer. For SDS PAGE, Novex™ 16% or 18% Tris-glycine gels were loaded with 5 μg protein per lane and run at 200 V for 1 h under denaturing conditions in SDS-Tris-glycine buffer. The gels were stained with Instant Blue protein stain (Abcam).

Size-exclusion (SEC) and heparin affinity chromatography SEC was performed using a Superose 6 10/300 GL column controlled by an ÄKTA UPC 10 FPLC system (GE Healthcare). Elution by 10 mM PBS, pH 7.4 was carried out at a flow rate of 0.5 mL/min.

For heparin affinity chromatography, HiTrap-HP column (GE Life Sciences, #17040601) was used. The column was equilibrated with 5 volumes of buffer A (10 mM sodium phosphate buffer, pH 7.4). SAA solution (0.5 mL of 0.5 mg/mL total protein concentration) was filtered with a 0.2 micron filter, loaded onto the column, and incubated for 10 min. The flow-through fraction was eluted with 5 column volumes of buffer A, and the bound fraction was eluted with 10 volumes of buffer B (10 mM sodium phosphate buffer, 1.0 M NaCl, pH 7.4) using a linear gradient of 0 -100% buffer B.

Heparin binding monitored by ELISA Heparin from porcine intestinal mucosa was used. Stock solution containing 10 mg/mL heparin in d.d. H_2O was sterilized by filtering and stored at 4 ^oC for up to one month. SAA interactions with heparin were quantified using ELISA Microtiter wells following published protocols (5). The wells (Nunc Immunosorp plates, Sigma, #Z755273) were coated with heparin by incubating overnight at 4 °C with 100 μL of 0.1 mg/mL heparin in 50 mM PBS at pH 7.4. The control wells were incubated with 10 mM PB, 135 mM NaCl, 0.05% Tween (PBST) at pH 7.4 containing

2% BSA (BSA-PBST). The wells were rinsed thrice with PBST, blocked using 200 µL of 2% BSA-PBST for 1 h at 20 °C, and washed thrice with PBST. Serial dilutions of SAA in 1% BSA-PBST (100 µL per well) were added, the wells were incubated for 1 h at 20 °C, and were washed thrice to remove unbound protein. Bound SAA was detected using ELISA as follows. The wells were incubated at 20 ºC for 1 h with primary SAA antibody (rabbit monoclonal for mSAA from Thermofisher, #700830, mouse monoclonal for hSAA from Meridian Lifesciences, #H86177M) at 1:1000 dilution in 2% BSA-PBST, using 100 µL per well, and were washed thrice with PBST. Next, the secondary antibody (Kindle Biosciences digital anti-mouse #R1005 or digital anti-rabbit # R1006) at 1:1000 dilution in 0.5% BSA-PBST was added at 100 µL per well, the wells were incubated at 20 ºC for 1 h, and were washed thrice. Color development was achieved after incubation at 25 ºC for 15 min with 100 µL of 3,3´,5,5´-tetramentylbenzidine substrate (Fisher #34028). The reaction was stopped using 2 M H_2SO_4. Absorbance at 450 nm was measured using a plate reader (Tecan Infinite M1000). To determine protein binding to wells, the absorbance of control wells without heparin was subtracted from the data. The assays were repeated thrice, with free SAA as an internal control.

Dot blot for oxidized lipids For dot blot, 2.5 µL of sample containing 1.0 mg/mL SAA in standard buffer was spotted on a nitrocellulose membrane (#09-301-109, Fisher) and dried at 24 °C. The membranes were washed with water and blocked in 5% w/v nonfat dry milk in Tween-20 (Boston Bioproducts, #IBB-181) for 2 h at 24 °C. The membranes were incubated at 24 °C for 1 h with EO6 antibodies for oxidized phospholipids (Avanti Polar Lipids #EO6-16), which were diluted 1:1000 in 5% nonfat dry milk in TBS with Tween-20. To ascertain that the protein load was comparable in different dots, all samples were probed with primary SAA antibodies (rabbit monoclonal from Thermofisher, # 700830, for mSAA or mouse monoclonal from Meridian Lifesciences, #H86177M for hSAA), which were diluted 1:1000 in 5% nonfat dry milk, for 1 h at room temperature. The blots were washed thrice in TBS with Tween-20 and incubated at 24 °C for 1 h with digital secondary antibody for mouse (Kindle Bioscience, #R1005), and digital secondary antibody for rabbit (Kindle Bioscience, # R1006), which was diluted 1:5000 in TBS with Tween-20. The membranes were washed thrice with Tween-20, and the signal was developed with Tannon Western Plus ECL Substrate (Abclonal, #180-501). The membranes were imaged with Fisher iBright 1500 imager.

CD36 binding monitored by ELISA CD36 binding was quantified following modified published protocols (6). Recombinant CD36-His_6 construct, which contained the His-tagged extracellular ligand-binding domain of human CD36, was expressed in HEK293 cells and purified commercially (Sino Biologicals Inc., cat. #10752-1708H, lot #LC081A1607). This protein was added to a 96-well Ni-NTA-coated HisSorbTM plate (Qiagen). A 100 µL protein aliquot in the protein-binding buffer (20 mM Tris-HCl,

pH 6.8, 150 mM NaCl, 1.5 mM $MgCl_2$, 5% v/v glycerol, 0.5% w/v *n*-dodecyl-D-maltoside) was added at 1 µg protein per well, and the wells were incubated overnight at 4 °C with gentle rocking. Unbound protein was aspirated, and the wells were washed thrice at 22 °C with 200 µL per well of the ligand-binding buffer (PBS, 1 mM $MgCl_2$, 1 mM $CaCl_2$, 0.5% w/v *n*-dodecyl-D-maltoside, 0.2% w/v fatty acid-free BSA). Next, the ligands (which contained either mSAA, hSAA or LDL) in ligand-binding buffer were added in a total assay volume of 100 µL, and the wells were incubated for 90 min at 22 °C with gentle rocking. Unbound ligand was removed, and the wells were washed thrice with 150 µL of ice-cold wash buffer (PBS, 1 mM $MgCl_2$, 1 mM $CaCl_2$, 0.5% w/v octyl glucoside, 0.05% w/v BSA) followed by three washes with 1 X PBST. An appropriate primary antibody was added to the wells at 100 µL per well: anti-mSAA (rabbit monoclonal for mSAA from Thermofisher, #700830, or mouse monoclonal for hSAA from Meridian Lifesciences, #H86177M) was added at 1:1000 dilution in PBST containing 2% BSA; anti-apoB (Santa Cruz #sc-13538 HRP) was added at 1:3000 dilution. The wells were incubated for 1 h at 20 ºC followed by three washes with 1 X PBST. Next, 100 µL per well of the secondary antibody (Kindle Biosciences, anti-mouse #R1005 or digital anti-rabbit # R1006) was added at 1:1000 dilution in PBST containing 0.5% BSA. The wells were incubated at 20 ºC for 1 h and washed. The color was developed after 15 min incubation at 25 ºC with 100 µL of 3,3´,5,5´-tetramentylbenzidine substrate (Fisher #34028). The reaction was quenched with 2 M H_2SO_4. Absorbance at 450 nm was recorded using a plate reader (Tecan Infinite M1000).

The assay was validated by comparing CD36 binding to intact vs. oxidized LDL. Single-donor plasma of healthy human volunteers was obtained from the local blood bank in compliance with their rules and regulations; plasma LDL was isolated by KBr density ultracentrifugation in the density range 1.019–1.063 g/mL with approval of the Institutional Review Board. Oxidized LDL was prepared by incubating intact LDL (0.5 mg/mL apoB in 10 mM PB, pH 7.4) with 10 µM $CuSO_4$ at 37 ºC for 12 h. The reaction was terminated with 1 mM EDTA followed by buffer exchange for CD36 measurements, which were done in technical triplicates. For SAA-containing ligands, the assays were repeated in technical and biological triplicates, using free SAA as an internal control. The binding data are reported as averages of technical and biological triplicates with standard errors.

Lipolysis by sPLA$_2$ and free fatty acids (FFA) and phospholipid assays SAA:lipid complexes (0.2 mM lipids, total volume 1 mL) were incubated at 37 °C with 10 µg/mL $sPLA_2$ in 150 mM NaCl, 2 mM $CaCl_2$, 2 mM $MgCl_2$, 20 mM Tris HCl at pH 7.4 in the presence of 2.5 mg/mL essentially fatty acid free bovine serum albumin (BSA). Control samples without $sPLA_2$ were incubated under identical conditions. After 3 h incubation, the lipolysis was terminated by adding EDTA to a final concentration of 1 mM. The extent of lipolysis was assessed by measuring the released FFA using an FFA kit (EnzyChromTM, EFFA-100). Final concentration of FFA was determined after subtracting the control data from the sample data.

Following manufacturer's recommendations, the first calibration curve (linear regression, R=0.997) was created in a wider concentration range of the standard (0-1000 µM palmitic acid). Next, the second calibration curve (linear regression, R=0.994) was created in a narrower concentration range (0-100 µM palmitic acid), which covers the concentration range for SAA:lipid samples used in this study. The background absorbance (without $sPLA_2$) was subtracted from the data, and the second linear calibration curve was used to estimate the final concentration of FFA released by hydrolysis. Following albumin removal using affinity column (Hi-trap Blue HP column, Cytiva #7-0412-01), the hydrolyzed particles were re-isolated by density gradient centrifugation at 1.06–1.22 g/mL KBr. All assays were done in technical and biological triplicates.

Amyloid formation SAA samples (0.2-0.25 mg/mL protein) were incubated at 37 ^oC with gentle stirring at 100 r.p.m. for up to 48 h. Sample aliquots were taken at different time points for analysis. Formation of amyloid-like structure was monitored using thioflavin T (ThT) fluorescence emission, which increases upon binding to amyloid-like structure. ThT was added to aliquots containing 10 µM SAA at a final dye concentration of 20 µM, which was determined spectrophotometrically at 412 nm using extinction coefficient of 36,000 $M^{-1}\cdot cm^{-1}$. ThT emission spectra at 25 °C were collected at 450-550 nm using λ_{ex}=440 nm with 5 nm excitation and emission bandwidths. Emission of ThT in buffer was subtracted from the data. The integrated emission intensity was determined by peak integration from 450 nm to 500 nm, and was plotted as a function of time. Each experiment was performed in quadruplicate. As a control, ThT was incubated at 37 ^oC with heparin and MLVs alone (0.02 mg/mL heparin and 1000 µM lipid), and the emission was recorded for up to 48 h; no significant emission changes were detected. Therefore, the increase in emission observed during protein fibrillation resulted from ThT binding to the protein rather than lipid. To harvest amyloid, after incubation the samples were centrifuged at 20,000 *g* for 20 min and washed thrice with 10 mM PB at pH 7.4. Pellets were collected from the bottom fraction, resuspended in buffer, and used for further studies.

Dot blots using amyloid-specific conformational antibodies For dot blot analyses, 2.5 µL of sample containing 1.0 mg/mL SAA in standard buffer was spotted on a nitrocellulose membrane (Fisher, #09-301-109) and dried at 24 °C. The membranes were stained with Ponceau-S, washed, and blocked in 5% w/v nonfat dry milk in Tween-20 (Boston Bioproducts, #IBB-181) for 2 h at 24 °C. Next, the membranes were incubated at 24 °C for 1 h with either A11 antibody for amyloid oligomers (Millipore, #AB9234) or OC antibody for amyloid fibrils (Millipore, #AB2286); the antobodies were diluted 1:1000 in 5% nonfat dry milk in Tris buffer saline with Tween-20. To ascertain that the protein load was comparable in different dots, all samples were probed with primary mSAA antibody (rabbit monoclonal for mSAA from

Thermofisher, #700830), which was diluted 1:2000 in 5% nonfat dry milk, for 1 h at room temperature. The blots were washed thrice in TBS with Tween-20 and incubated at 24 °C for 1 h with digital secondary antibody for mouse (Kindle Bioscience, #R1005), and digital secondary antibody for rabbit (Kindle Bioscience, #R1006), which was diluted 1:5000 in TBS with Tween-20. The membranes were washed thrice with Tween-20, and the signal was developed with Tannon Western Plus ECL Substrate. The membranes were imaged with Fisher iBright 1500 imager.

Transmission electron microscopy (EM) For transmission EM of negatively stained samples, the samples were diluted to 0.05-0.1 mg/mL SAA or to ~0.05 mg/mL lipid, and a 4 µL clear drop was deposited onto grids. Fibril-containing samples were centrifuged, and a clear drop was deposited onto grids. The grids were washed with d.d. water and stained with 1% uranyl acetate. Electron micrographs were collected under low-dose conditions at 45,000–65,000 x magnification using a CM12 transmission electron microscope (Philips Electron Optics, the Netherlands) at 100 kV equipped with a Tietz 2K x 2K CCD camera (TVIPS, Gauting, Germany).

Circular Dichroism (CD) spectrometry CD data were recorded using a Jasco J-1500 spectro-polarimeter (Jasco, Japan) to monitor protein secondary structure and thermal stability. Far-UV CD spectra were recorded at 190-250 nm from solutions containing 0.1 mg/mL SAA in standard buffer; the spectra recorded at 25° C and 37 °C closely overlapped. CD spectra at 25 °C are shown throughout the paper to match FT IR spectra (which could only be recorded at room temperature). Melting data were recorded at 222 nm to monitor secondary structural unfolding and refolding during sample heating and cooling at a constant rate of 70 °C/h. Buffer baselines were subtracted from the data; the results were normalized to the protein concentration and reported as molar residue ellipticity, [Θ]. Helical content was estimated based on the measured value of [Θ] at 222 nm: α-helix % = ($[\theta_{222}]$ – 3,000)/(-36,000 – 3,000). The results were in excellent agreement with the values determined using CD secondary structural analysis using BeStSel software (7) freely available via the webserver https://bestsel.elte.hu.

Fourier Transform Infrared (FT IR) spectroscopy The spectra were recorded at room temperature using a Jasco ATR-pro penta FTIR-6600 spectrometer (Jasco, Japan) with a MCT-6000PV, MCT(PV) detector equipped with attenuated total reflection accessory. Prior to data collection, the samples were concentrated to ~2 mg/mL; 5 µL of protein was placed on the Ge crystal of the ATR accessory. Measurements were performed as an accumulation of 64 scans with 2 cm^{-1} resolution over a wavenumber range of 1,000–4,000 cm^{-1}. The spectra were recorded with 8 mm aperture and 10 kHz scanner velocity. Atmospheric compensation and baseline correlation were executed using the

atmospheric compensation algorithm within the Jasco software. Individual secondary structural components were determined by spectral deconvolution using Jasco spectral manager Version 2.

Fluorescence spectroscopy Intrinsic Trp fluorescence was monitored at 25 °C using a Jasco FP-8500 spectrofluorimeter (Jasco, Japan). The samples contained 0.1 mg/mL SAA. The excitation wavelength was λ_{ex}=295 nm and the emission was recorded at 310 – 450 nm with 5 nm excitation and emission slit widths. The wavelength of maximal fluorescence, λ_{max}, was determined from the emission peak with an accuracy of ±2 nm or better.

Fluorescence anisotropy was measured at 37 ^{o}C with a Jasco FP-8500 spectrometer. The measurements were taken at λ_{ex}=295 nm for tryptophan and at the emission maximum. The anisotropy (r) was calculated according to the equation: $r = I_{vv}\text{-}G \times I_{vh}/I_{vv}+2G \times I_{vh}$

Here, I is the intensity of the emitted light; the first subscript refers to the vertical (v) or horizontal (h) component of the dielectric vector of the excitation, and the second subscript refers to those for emission; the grating factor $G = I_{hv} / I_{hh}$ corrects for non-equal sensitivity of the instrument to horizontally and vertically polarized light. G-factor correction is pre-programmed in the Jasco software, and the value is corrected for each sample.

Time-resolved fluorescence was measured using Horiba Deltaflex modular fluorescence lifetime system at λ_{ex}= 285 nm. The emission was collected through a WG 305 long-pass filter and a U-330 band-pass filter to remove scattered light. Fluorescence decay curves were recorded in 1000 channels with a 0.1 ns/channel resolution. The instrument response factor was determined using Ludox solution; p-terphenyl in absolute ethanol was used as a lifetime reference (τ = 1.05 ns). The results were subjected to global analysis using data fitting with single-, double- or triple-exponential decay functions. The best results (lowest χ^2 values) were obtained with double-exponential data fitting and are reported in supplementary Tables 5 and 9.

Statistical analysis All key experiments in this study were repeated using five technical and three biological replicates for mSAA, and three technical and two biological replicates for hSAA; in biological replicates different protein batches were used. The data are presented as mean values ±SEM. Data analyses were performed using Graphpad Prism 9.0, version 9.5.1 (San Diego, CA) and Origin, Version 7.0383 (Originlab corporation, MA). The statistical significance of the difference between two groups of measurements was determined using unpaired t-test. A value of $p < 0.05$ was considered significant.

SUPPLEMENTAL TABLES S1 – S9

Lipid	Chemical structure	T_c, °C
1-palmitoyl-2-oleoyl-sn-glycero-3-phosphocholine (POPC)	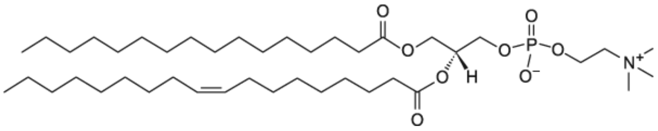	-2
1-palmitoyl-2-oleoyl-sn-glycero-3-phospho-ethanolamine (POPE)	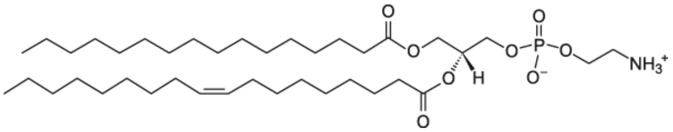	25
1-palmitoyl-2-oleoyl-sn-glycero-3-phospho-(1'-rac-glycerol) (POPG)	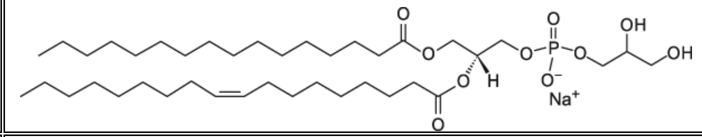	-2
Sphingomyelin (SM)	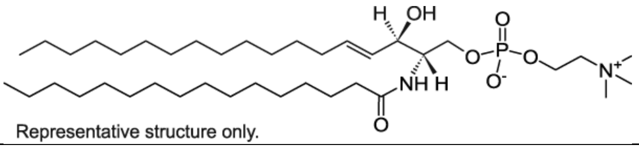	38 *
1-palmitoyl-2-oleoyl-sn-glycero-3-phospho-L-serine (POPS)	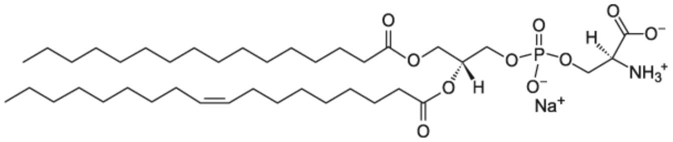	14
1-palmitoyl-2-oleoyl-sn-glycero-3-phosphate (POPA)	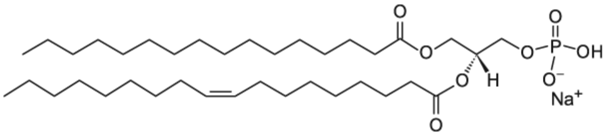	28
L-α-phosphatidylinositol (PI)	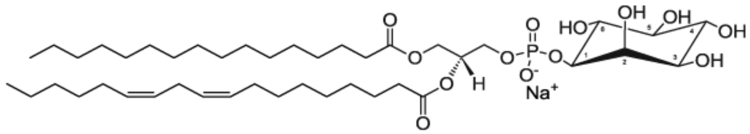	N/A
1',3'-bis[1,2-dimyristoyl-sn-glycero-3-phospho]-glycerol Cardiolipin (CL)	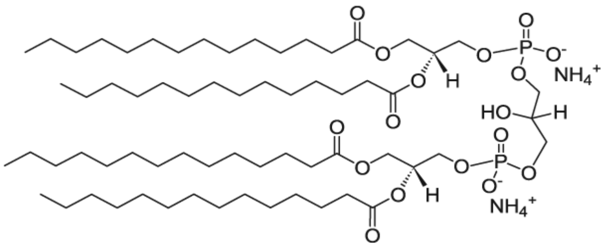	62

Table 1 Phospholipids explored in the current study. Chemical structures and the values of the major thermotropic gel-to-fluid phase transition temperature, T_c, in hydrated stacked bilayers are from Avanti Polar Lipids, https://avantilipids.com/. T_c of SM is from (8); T_c of PI is not provided, and pure PI does not show a phase transition above 0 °C (9).

Phospholipid	Final lipid, mg
POPC	0.21 ± 0.010
POPE	0.54 ± 0.027
POPG	0.55 ± 0.027
SM	0.65 ± 0.032
PI	0.53 ± 0.026
POPA	0.61 ± 0.030
POPS	0.68 ± 0.034
CL	0.12 ± 0.006

Table 2 Lipid quantification in SAA complexes with model phospholipids. In all preparations the initial protein:lipid molar ratio was 1:100. SAA was incubated with lipids for 2 h at 37 ^{o}C, and SAA:lipid complexes were isolated by density gradient centrifugation. 1 mL of sample containing 0.5 mg/mL protein was delipidated and the dry weight of lipid was measured. Mean values of three independent measurements with their standard errors are listed per 0.5 mg protein.

Sample	α-helix, %	β-sheet, %
free SAA	13 ± 1.6	15 ± 1.2
SAA:POPC	45 ± 2.2	18 ± 1.4
SAA:SM	35 ± 1.7	20 ± 1.6
SAA:POPE	35 ± 1.7	30 ± 2.4
SAA:POPG	33 ± 1.6	31 ± 2.5
SAA:POPS	31 ± 1.5	28 ± 2.2
SAA:POPA	22 ± 1.1	28 ± 2.4
SAA:PI	34 ± 1.7	28 ± 2.8
SAA:CL	38 ± 1.9	30 ± 2.4
SAA:lysoPC	38 ± 2.5	26 ± 2.9
SAA:lysoPE	22 ± 2.3	31 ± 2.4
SAA:lysoPS	21 ± 2.4	33 ± 2.6
SAA:OA	32 ± 2.7	22 ± 2.8

Table 3 Secondary structural content in SAA lipid-free or in complexes with model lipids. The α-helix content was determined by far-UV CD at 22-37 °C using the molar residue ellipticity at 222 nm, $[\theta_{222}]$, as $\alpha\% = ([\theta_{222}] - 3{,}000)/(-36{,}000 - 3{,}000)$. Very similar values were obtained using CD analysis by BeStSel software [7]. Unless otherwise stated, the β-sheet content was obtained at 22 °C by FT IR spectral deconvolution using Jasco software. Tabulated values represent an average of triplicate measurements from two independent reconstitution experiments with the standard errors. For complexes with lysoPL and OA, the α-helix and β-sheet content was obtained by CD analysis using BeStSel with ~5% accuracy.

Sample	λ_{max}, nm	anisotropy
free SAA	350	0.063
SAA:POPC	340	0.095
SAA:SM	345	0.093
SAA:POPE	336	0.105
SAA:POPG	335	0.108
SAA:POPS	335	0.118
SAA:POPA	338	0.112
SAA:PI	338	0.102

Table 4 Trp emission and fluorescence anisotropy parameters for lipid-free SAA and SAA complexes with model lipids. The values represent an average of five measurements from two independent reconstitution experiments. Wavelength of maximal emission, λ_{max}, was determined with an accuracy of ±2 nm or better from the Trp emission spectra such as those shown in Fig. 1g. Anisotropy values were determined as described in Methods with STD of ± 0.005.

Sample	τ1, ns	τ2, ns	a1	a2	<τ>, ns	X^2
free SAA	1.12 ± 0.07	2.7 ± 0.23	0.32 ± 0.02	0.68 ± 0.06	1.09	1.1
SAA:POPC	1.09 ± 0.06	3.4 ± 0.13	0.2 ± 0.05	0.8 ± 0.03	1.47	1.01
SAA:SM	1.13 ± 0.05	3.7 ± 0.18	0.2 ± 0.07	0.8 ± 0.05	1.59	1.18
SAA:POPE	0.98 ± 0.05	4.1 ± 0.18	0.8 ± 0.07	0.2 ± 0.05	0.80	1.37
SAA:POPG	0.95 ± 0.03	4.2 ± 0.21	0.8 ± 0.07	0.2 ± 0.05	0.80	1.30
SAA:POPS	0.93 ± 0.07	4.5 ± 0.25	0.85 ± 0.04	0.15 ± 0.07	0.73	1.17
SAA:POPA	0.97 ± 0.03	4.1 ± 0.22	0.85 ± 0.03	0.15 ± 0.03	0.72	1.0
SAA:PI	0.98 ± 0.06	3.9 ± 0.22	0.82 ± 0.04	0.18 ± 0.04	0.75	1.2
SAA:CL	0.91 ± 0.04	3.5 ± 0.20	0.8 ± 0.03	0.2 ± 0.05	0.71	1.1

Table 5 Trp fluorescence lifetime parameters for lipid-free SAA and SAA complexes with model lipids. The data, which were recorded as described in Methods, were approximated using double-exponential decay function to determine Trp fluorescence lifetimes, τ1 and τ2, and fractional amplitudes, a1 and a2. The average lifetime represents weighted average, $<\tau> = \tau1 \cdot a1 + \tau2 \cdot a2$. Values represent averages of triplicate measurements from two independent reconstitution experiments.

Organ lipid extract	Final dry weight, mg
brain	1:28 ± 0.064
heart	1:3 ± 0.065
liver	1:25 ± 0.075

Table 6 Lipid quantification in SAA complexes with organ lipid extracts. In all preparations the initial amount of SAA was 0.5 mg/mL (43 µM) and the initial lipid concentration was 2 mg/mL (1:4 protein:lipid wt/wt). SAA was incubated with lipids for 12 h at 37 ^oC; SAA:lipid complexes were isolated by density gradient centrifugation. 1 mL of sample was delipidated and the dry weight of lipid was measured. Averages of three independent measurements are listed with their standard errors.

Sample	α-helix, %	β-sheet, %
SAA:brain lipids	35 ± 1.6	22 ± 2.4
SAA:heart lipids	39 ± 1.9	11 ± 1.2
SAA:liver lipids	26 ± 1.3	30 ± 2.4
free SAA	13 ± 1.6	15 ± 1.2

Table 7 Secondary structural content in lipid-free SAA and in SAA complexes with model lipids. The α-helix content was determined by far-UV CD from the value of $[\Theta_{222}]$ and the β-sheet content was obtained using FT IR spectral deconvolution. Tabulated values represent an average of triplicate measurements from two independent reconstitution experiments with the standard errors.

Sample	λ_{max}, nm	anisotropy
SAA:brain	335	0.118
SAA:heart	335	0.110
SAA:liver	340	0.138

Table 8 Steady-state Trp fluorescence and fluorescence anisotropy parameters for SAA complexes with organ lipid extracts. The values represent an average of five measurements from two independent reconstitution experiments. Wavelength of maximal emission, λ_{max}, was determined with ±2 nm accuracy from the Trp emission spectra (Fig. 2h). Anisotropy values were determined with STD of ± 0.005.

Sample	τ1, ns	τ2, ns	a1	a2	<τ>, ns	X^2
free SAA	1.12 ± 0.07	2.7 ± 0.23	0.32 ± 0.02	0.68 ± 0.06	1.09	1.1
SAA:brain	0.92 ± 0.07	4.7 ± 0.23	0.39 ± 0.02	0.61 ± 0.06	1.61	1.1
SAA:heart	0.93 ± 0.04	4.87 ± 0.22	0.44 ± 0.03	0.56 ± 0.04	1.57	1.3
SAA:liver	0.98 ± 0.07	5.01 ± 0.25	0.62 ± 0.06	0.38 ± 0.05	1.26	1.1

Table 9 Trp fluorescence lifetime parameters for lipid-free SAA and SAA complexes with organ lipid extracts. The parameters are described in supplemental Table 5 legend. Average values from triplicate measurements of two independent reconstitution experiments are shown with their standard errors.

SUPPLEMENTAL FIGURES 1-9

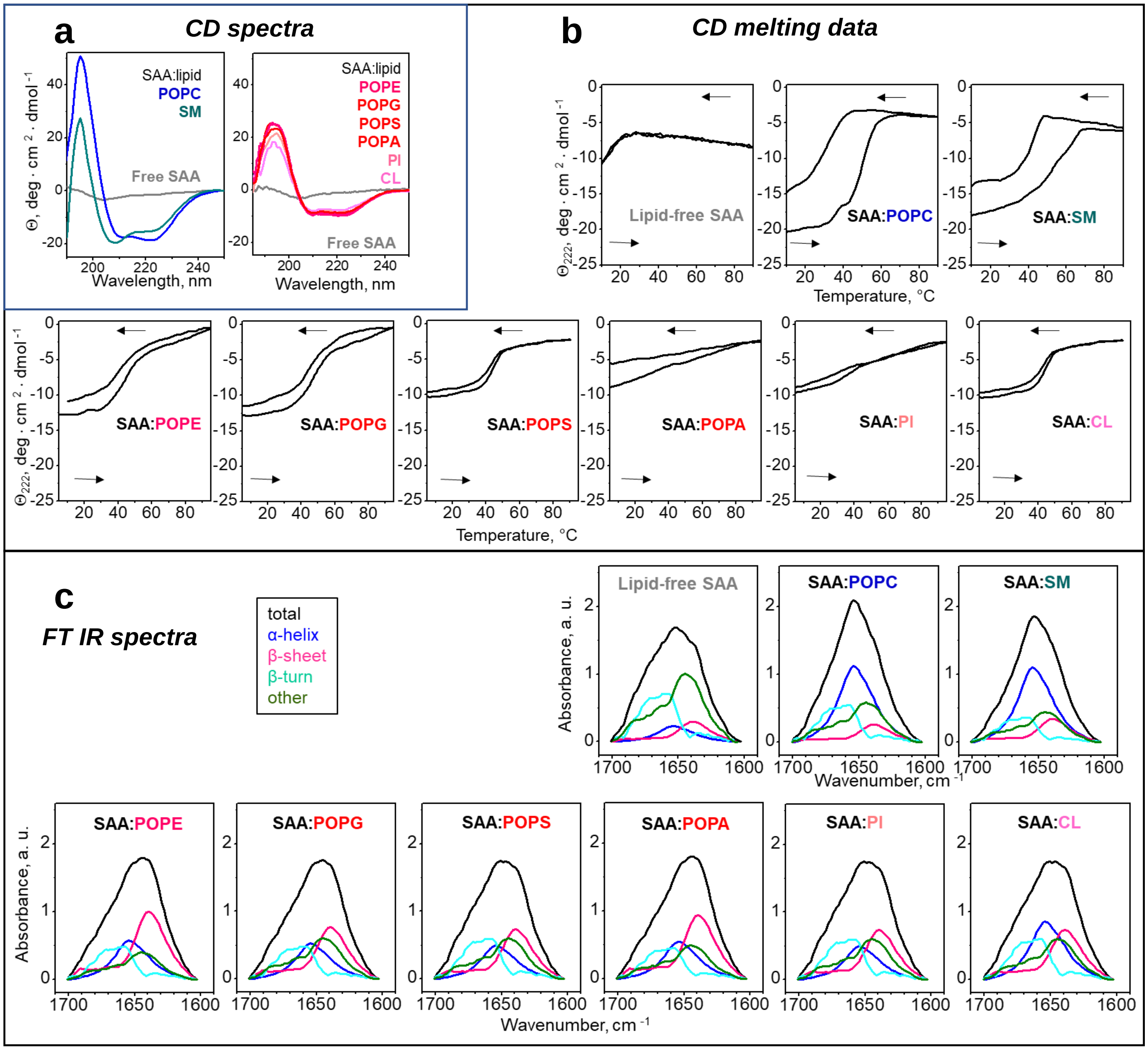

Supplemental Fig. 1 Secondary structure and thermal stability of SAA:lipid complexes formed upon solubilization of model lipids at pH 7.4. The complexes were prepared as in Fig. 1 (**A**) Far-UV circular dichroism (CD) spectra, (**B**) CD heating/cooling data, and (**C**) Fourier transform infrared (FT IR) spectra were recorded as described in Methods. Data for free SAA, SAA:POPC and SAA:PS are also shown in Fig. 1E. Experimental conditions (described in Methods) and color-coded as in Figure 1; lipid-free SAA is shown for comparison. Arrows in (B) show directions of the temperature changes. Panel C shows FT IR spectra (black lines) and their secondary structural analysis by spectral deconvolution (color lines).

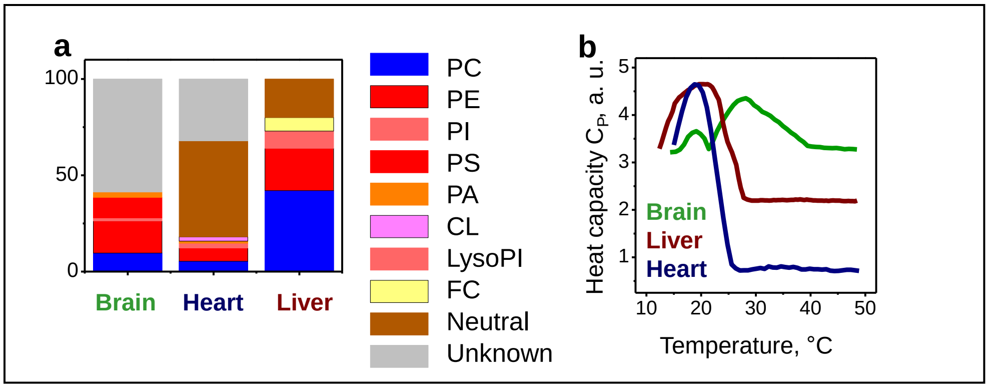

Supplemental Fig. 2 Composition and characterization of organ lipid extracts used in the current study. Extracts from bovine brain (green), heart (navy) and liver (wine) were from Avanti Polar Lipids. (**a**) Lipid composition supplied by the manufacturer. (**b**) Differential scanning calorimetry data recorded during heating of emulsions of lipid extracts. Experimental details are described in Methods.

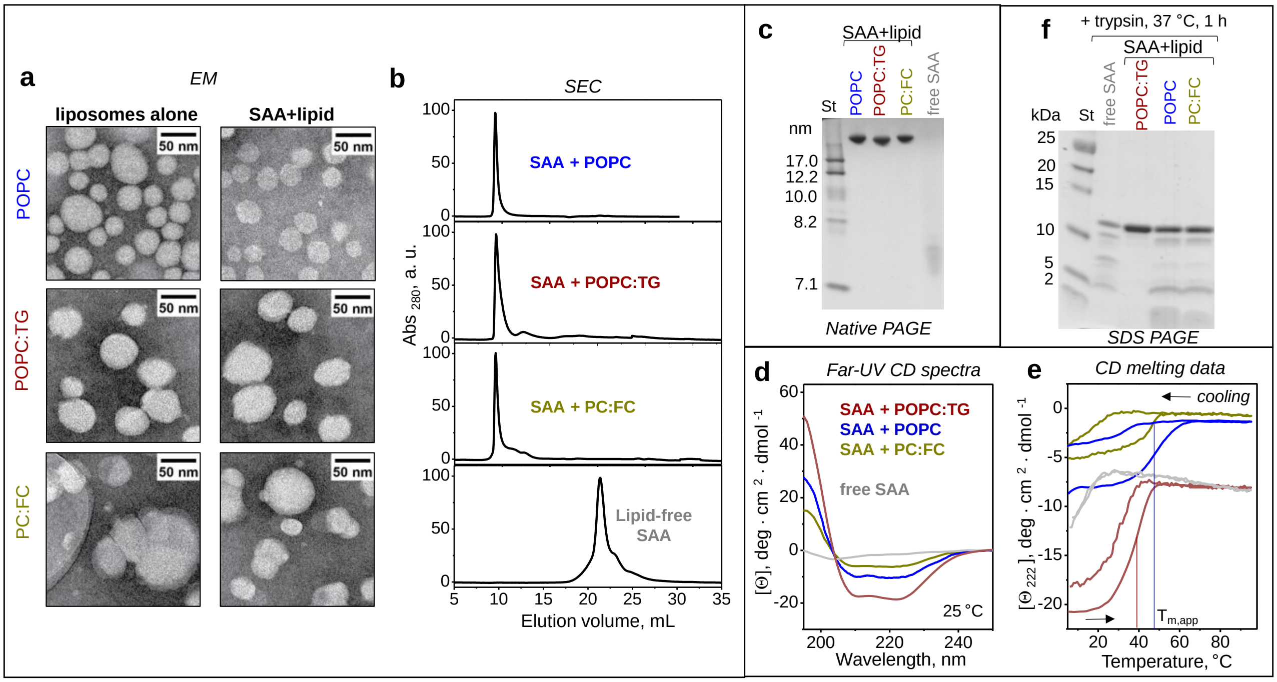

Supplemental Fig. 3 SAA+lipid complexes formed upon adsorption of mSAA on the surface of SUV or small lipid emulsions without their remodeling. SUVs of POPC (blue) or small emulsions of mixtures of POPC:TG (brown) or PC:FC (exosomal liposome, olive) were prepared by sonication as described in Methods. These SUVs/small emulsions were incubated for 3 h at pH 7.4, 37 °C with SAA (1:100 mole/mole protein:lipid) and the total incubation mixture was explored. (**a**) EM images of freshly made small lipid particles alone or after incubation with SAA (SAA+lipid). (**b**) SEC profiles show that all protein in the SAA+lipid samples adsorbed to the lipid surface; free SAA is shown for comparison. (**c**) Native PAGE of SAA+lipid complexes. (**d**) Far-UV CD spectra and (**e**) the heating / cooling data of SAA+lipid samples; the directions of temperature changes and the apparent melting temperatures, $T_{m,\ app}$, are indicated in panel E. (**f**) Limited proteolysis of SAA+lipid complexes monitored by SDS PAGE. The complexes have been incubated with trypsin for 1 h at 37 °C as described in Methods. Free SAA is shown for comparison.

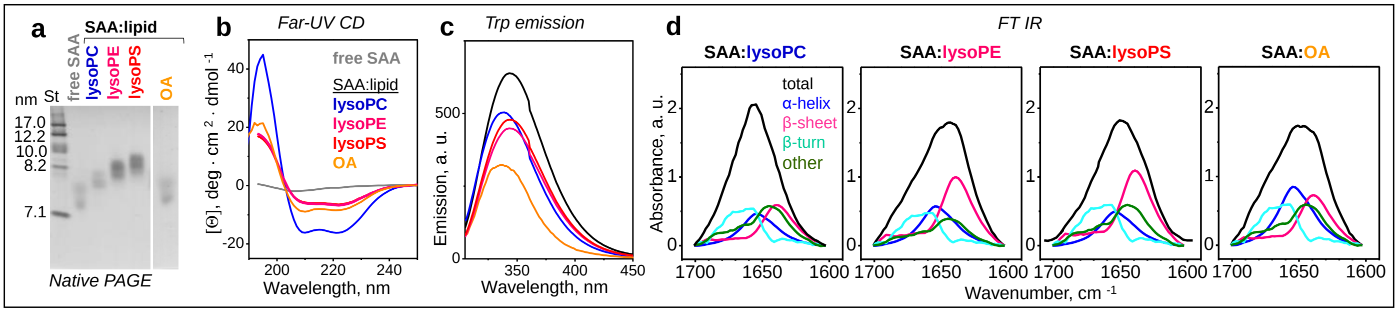

Supplemental Fig. 4 Structural properties of SAA complexes with lyso-phospholipids and oleic acid. (**a**) Native PAGE, spliced image is separated by white line. (**b**) far-UV CD spectra, (**c**) Trp emission spectra, and (**d**) FT IR spectra of SAA complexes with lysoPC (blue), lysoPE, lysoPS (red) or OA (orange). The complexes were prepared by spontaneous solubilization as described in Methods.

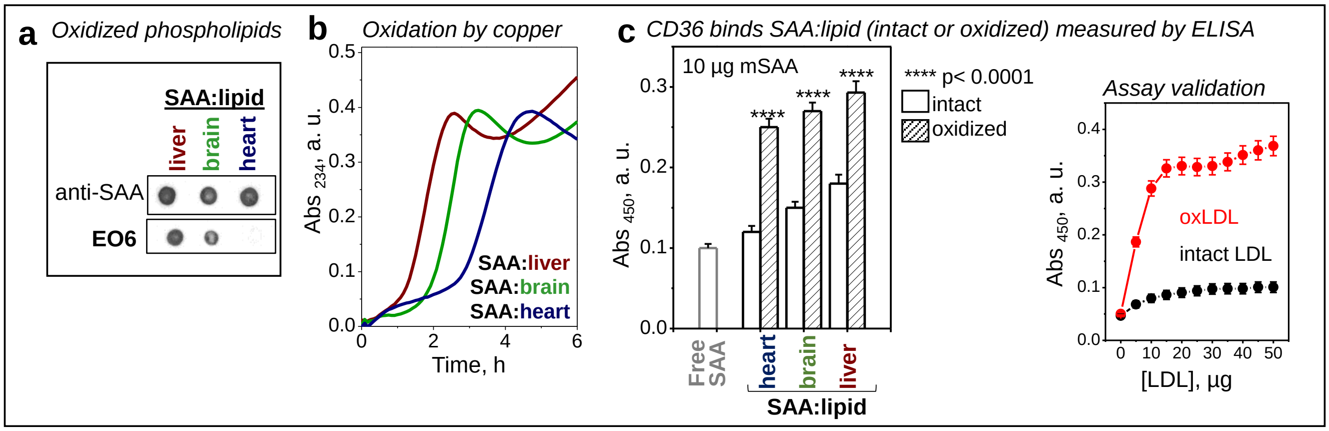

Supplemental Fig. 5 CD36 binding to SAA:lipid complexes is augmented by their oxidation. The complexes with were formed upon solubilization by mSAA of large lipid emulsions prepared from mammalian lipid extracts, as shown in Figure 2 and described in Methods. (**a**) Dot blot of intact SAA:lipid complexes using EO6 antibody detects more endogenous oxidized phospholipids in complexes with heart and brain vs. liver lipids; anti-SAA is used as a control. (**b**) Time course of copper-induced phospholipid oxidation in SAA:lipid complexes monitored by absorbance at 234 nm for conjugated dienes formation. (**c**) ELISA using the immobilized ligand-binding domain of CD36 shows the binding for intact and for copper-oxidized SAA:lipid complexes (open and closed bars); data for free SAA is shown for comparison. The values are mean of three independent measurements ±SEM. For each complex, binding of oxidized vs. intact complexes is compared. Binding of oxidized but not intact LDL validates the assay (right panel).

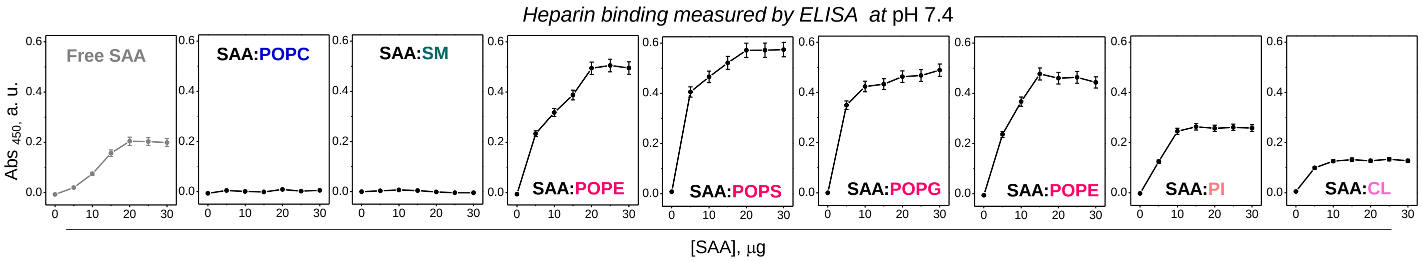

Supplemental Fig. 6 Heparin binding to SAA:lipid complexes is lipid dependent. Complexes of mSAA with model lipids were prepared by solubilization, and their binding to immobilized heparin was monitored by ELISA as described in Methods. Binding data for free SAA are shown for comparison. Average values for technical and biological triplicates with associated standard errors are shown.

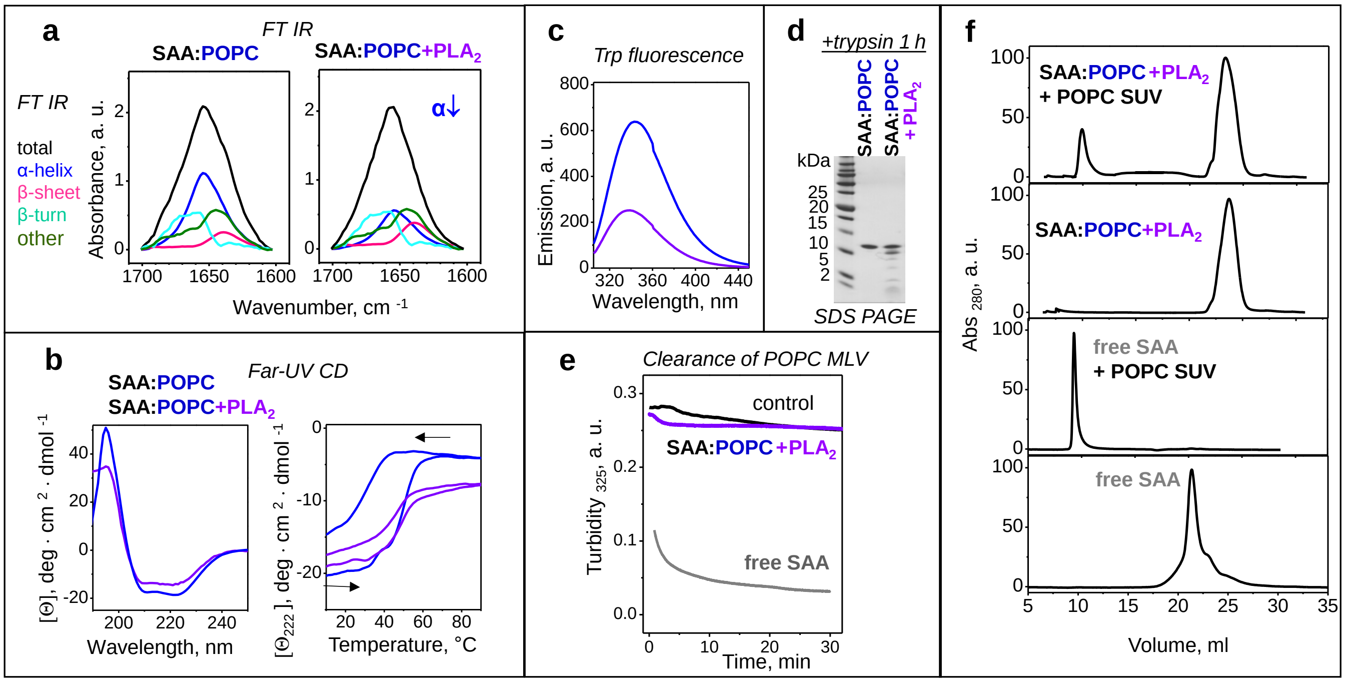

Supplemental Fig. 7 Structural remodeling of SAA:POPC complexes by sPLA$_2$ at pH 7.4 and their lipid binding properties. The complexes were either intact (SAA:POPC, blue) or incubated with sPLA$_2$ at 37 °C, pH 7.4 for 3 h (SAA:POPC+PLA$_2$, violet); selected data for free SAA (gray) are shown for comparison. (**a**) FT IR spectra, (**b**) far-UV CD spectra and the heating/cooling data, and (**c**) Trp emission spectra. (**d**) SDS PAGE of the complexes that have been incubated with trypsin for 1 h at 37 °C. (**e**) Clearance kinetics of POPC MLVs at 37 °C, pH 7.4 monitored by turbidity; MLVs alone (no SAA) provide a negative control. (**f**) SEC profiles of SAA:POPC+PLA$_2$ alone and together with POPC SUV probe their adhesion. Similar profiles for free SAA are shown as a positive control.

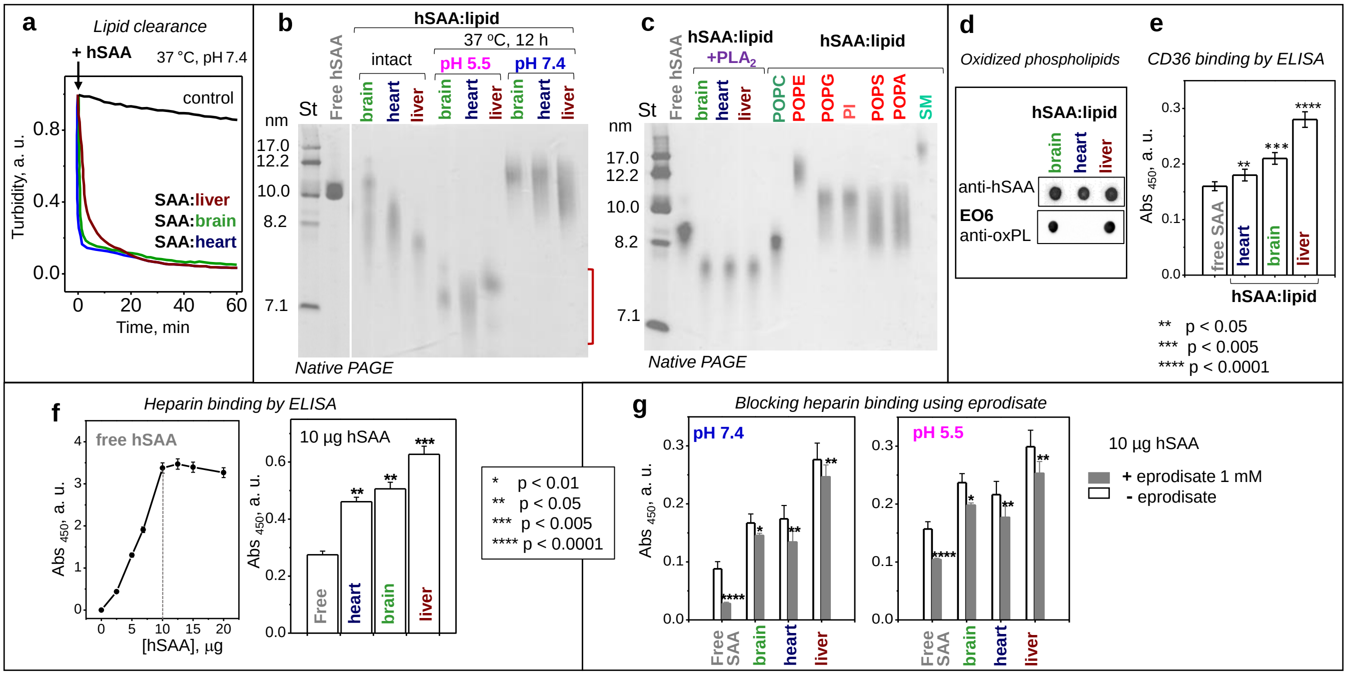

Supplemental Fig. 8 Human SAA:lipid complexes: formation, structural and functional properties. (**a**) Clearance kinetics by hSAA of large emulsions of organ lipids at 37 °C, pH 7.4 monitored by turbidity (colored lines); lipids alone (black) are a negative control. For further studies, hSAA:lipid complexes were prepared and isolated by density as described in Methods. (**b, c**) Native PAGE of hSAA complexes with organ or model lipids. The spliced image is shown in white line. Complexes with organ lipid extracts were: i) intact; ii) after 12 h incubation at 37 °C at pH 5.5 or 7.4; or iii) incubated with $sPLA_2$ for 3 h at 37 °C, pH 7.4. Complexes with model lipids were intact. Unlike mSAA, hSAA formed particles of different sizes with lipid extracts from different organs. Differences in migration of free hSAA reflect batch-to-batch variations. Red bracket indicates migration range of lipid-poor SAA. (**d, e**) CD36 binding to complexes of hSAA with organ lipids is augmented by lipid oxidation. Dot blot shows that, like mSAA:lipid (supplemental Fig. 5), intact hSAA:lipid complexes contain more oxidized phospholipids in liver and brain vs. heart extracts (D). ELISA using immobilized extracellular domain of CD36 shows that the binding of hSAA to CD36 shows a modest increase in order: free SAA ≤ SAA:heart < SAA:brain < SAA:liver, similar to that observed for mSAA (supplemental Fig. 5). (**f**) ELISA using immobilized heparin shows a dose-dependent increase in binding that levels off at ~10 µg hSAA. ELISA using immobilized heparin and 10 µg hSAA shows that heparin binding increases in order: free SAA < SAA:heart ≤ SAA:brain < SAA:liver. (**G**) ELISA using immobilized heparin and 10 µg hSAA without or with 1 mM eprodisate at pH 7.4 and 5.5 shows that the drug fully blocks heparin binding for free hSAA but only partially for hSAA:lipid, similar to mSAA (Fig. 3e).

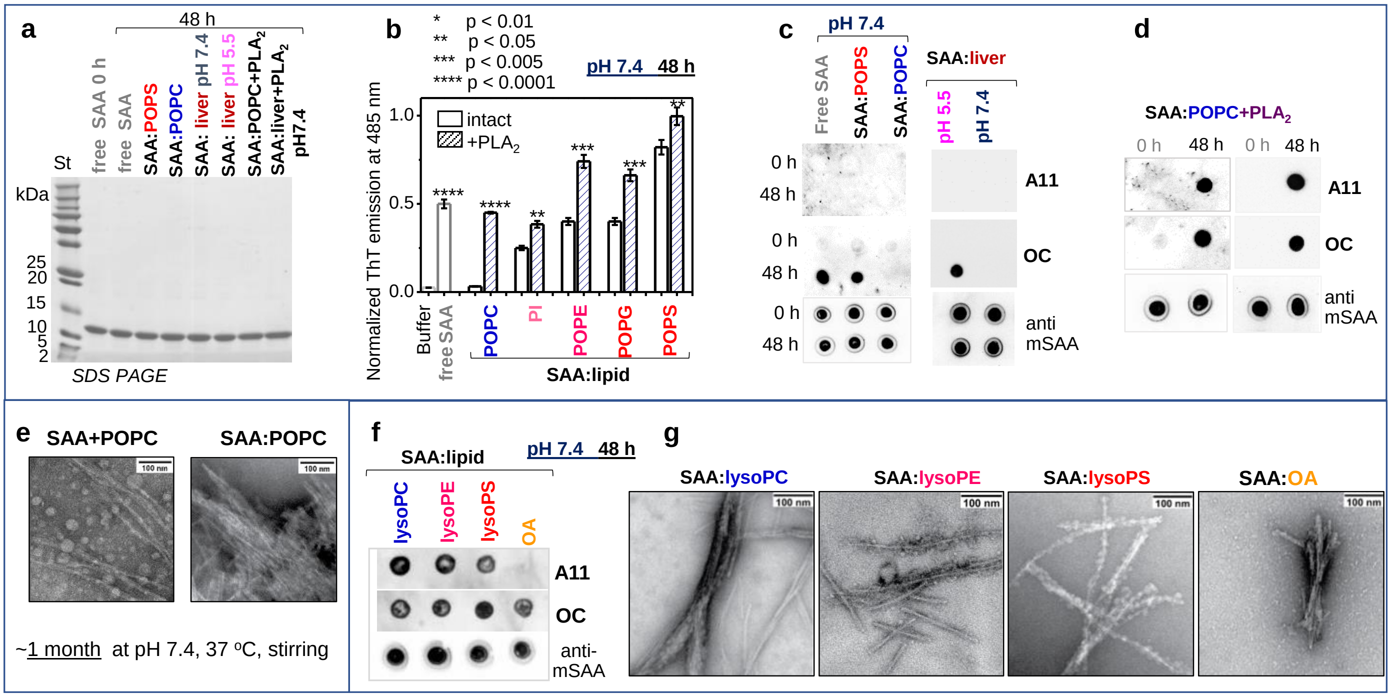

Supplemental Fig. 9 Sample characterization upon incubation of SAA:lipid complexes to explore the effects of lipids, pH and lipolysis on amyloid formation. (**a**) SDS PAGE of samples that have been incubated for 48 h under amyloid-promoting conditions (37 °C with stirring); lipid-free SAA before and after incubation is shown as a control. (**b**) Summary of ThT emission data recorded of SAA:lipid complexes that have been incubated under amyloid-promoting conditions at pH 7.4; the complexes were either intact or have been hydrolyzed with $sPLA_2$ (+PLA_2) and re-isolated prior to incubation. ThT intensity of intact vs. PLA_2-treated samples is compared. (**c, d**) Dot blots using A11 and OC antibodies for amyloid oligomers and fibrils, respectively, and anti-mSAA (as a control) for samples that were intact (0 h) or have been incubated for 48 h under amyloid-promoting conditions. (**e**) Electron micrographs of SAA+POPC samples (formed by SAA adsorption to POPS SUV, supplemental Fig. 3) and SAA:POPC (formed by SAA solubilization of POPC MLV, Fig. 1) that have been incubated for ~1 month under amyloid-promoting conditions. (**f**) Dot blot of SAA complexes with lyso-phospholipids or oleic acid (OA) after 48 h incubation at amyloid-promoting conditions, pH 7.4. (**G**) Electron micrographs of the samples from panel f. Bar size in panels e and g is 100 nm.

SUPPLEMENTAL REFERENCES

1. Kollmer M, Meinhardt K, Haupt C, Liberta F, Wulff M, Linder J, et al. Electron tomography reveals the fibril structure and lipid interactions in amyloid deposits. Proc Natl Acad Sci U S A. 2016; 113(20):5604–9.

2. Shimon MB, Shapira S, Seni J, Arber N. The Big Potential of Small Particles: Lipid-Based Nanoparticles and Exosomes in Vaccination. Vaccines (Basel). 2022 Jul 13;10(7):1119.

3. Mortimer BC, Simmonds WJ, Joll CA, Stick RV, Redgrave TG. Regulation of the metabolism of lipid emulsion model lipoproteins by a saturated acyl chain at the 2-position of triacylglycerol. J Lipid Res. 1988; 29(6):713–20.

4. Folch J, Lees M, Sloane Stanley GH. A simple method for the isolation and purification of total lipides from animal tissues. J Biol Chem. 1957; 226(1):497–509.

5. Fraser PE, Darabie AA, McLaurin JA. Amyloid-beta interactions with chondroitin sulfate-derived monosaccharides and disaccharides. implications for drug development. J Biol Chem. 2001;276(9):6412–9.

6. Martin CA, Longman E, Wooding C, Hoosdally SJ, Ali S, Aitman TJ, et al. Cd36, a class B scavenger receptor, functions as a monomer to bind acetylated and oxidized low-density lipoproteins. Protein Sci. 2007 Nov;16(11):2531–41.

7. Micsonai A, Moussong É, Wien F, Boros E, Vadászi H, Murvai N, et al. BeStSel: webserver for secondary structure and fold prediction for protein CD spectroscopy. Nucleic Acids Res. 2022; 50(W1):W90–8.

8. Arsov Z, González-Ramírez EJ, Goñi FM, Tristram-Nagle S, Nagle JF. Phase behavior of palmitoyl and egg sphingomyelin. Chem Phys Lipids. 2018; 213:102–10.

9. Peng A, Pisal DS, Doty A, Balu-Iyer SV. Phosphatidylinositol induces fluid phase formation and packing defects in phosphatidylcholine model membranes. Chem Phys Lipids. 2012; 165(1):15–22.

RESOURCES TABLES

Antibodies

Target antigen	Vendor	Catalog #
Murine SAA	Thermofisher	700830
Human SAA	Meridian Lifesciences	H86177M
apoB	Santacruz	sc-13538 HRP
A11	Millipore	AB9234
OC	Millipore	AB2286
Oxidized phospholipids	Avanti Polar Lipids	330001S
Anti-rabbit	Kindle Bioscience	R1006
Anti-mouse	Kindle Boscience	R1005

Assays

Description	Source	Catalog #
Phospholipid	Fisher Scientific	EPLP-100
Triglyceride	Fisher Scientific	ETGA-200
Free fatty acid	Fisher Scientific	EFFA-100

Materials sources

No	Chemical	Vendor	Catalog #
1	egg-PC, L-α-phosphatidylcholine	Avanti Polar Lipids	840051C
2	1-palmitoyl-2-oleoyl-glycero-3-phosphocholine	Avanti Polar Lipids	850457C
3	1-palmitoyl-2-oleoyl-sn-glycero-3-phosphoethanol (sodium salt)	Avanti Polar Lipids	840514C
4	1-palmitoyl-2-oleoyl-sn-glycero-3-phospho-(1'-rac-glycerol) (sodium salt)	Avanti Polar Lipids	840457C
5	1-palmitoyl-2-oleoyl-sn-glycero-3-phospho-L-serine (sodium salt)	Avanti Polar Lipids	840034C
6	L-α-phosphatidylinositol (Soy) (sodium salt)	Avanti Polar Lipids	840044C
7	L-α-phosphatidic acid (Egg, Chicken) (sodium salt)	Avanti Polar Lipids	840101C
8	Sphingomyelin (Egg, Chicken)	Avanti Polar Lipids	860061C
9	1,1’,2,2’-tetramyristoyl cardiolipid	Avanti Polar Lipids	710332
10	Brain lipid extract, total (porcine)	Avanti Polar Lipids	131101C
11	Heart lipid extract, total (Bovine)	Avanti Polar Lipids	171201C

12	Liver lipid extract, total (Bovine)	Avanti Polar Lipids	181104C
13	Triolein	Avanti Polar Lipids	870110
14	16:0 lyso PE	Avanti	855675
15	16:0 Lyso PC	Avanti	855675
16	16:0 Lyso PS	Avanti	858142
17	Sodium oleate	Sigma	O7501
18	Exosome liposomes PC:cholesterol (70:30 mole:mole)	Sigma	CEP-500
19	Trypsin from bovine pancreas	Sigma	T1426
20	Phenylmethanesulfonyl fluoride solution	Sigma	93482
21	Thioflavin T	Sigma	T3516
22	3,3´,5,5´-tetramentylbenzidine (TMB)	Fisher	34028
23	Heparin sodium salt from porcine mucosa	Sigma	H4784
24	Recombinant human SAA1	Peprotech	300-53
25	Phospholipase A2 from honey bee venom	Sigma	P9279,
26	Tannon high-sigECL Western blotting substrate	Abclonal	180-501
27	Instant blue Coomassie stain	Abcam	Ab119211
28	Human Cd36/SCARB3 protein (His tag)	Sino Biological	10752-H08H
29	BSA, essentially fatty acid free	Sigma	A4612
30	10X TBST, Tris Buffered Saline-Tween (10X, with 1% Tween-20, pH 7.4)	Boston Bioproducts	IBB-180
31	Phosphate Buffered Saline-Tween (10X, with 0.5% Tween-20, pH 7.4)	Boston Bioproducts	IBB-171
32	Sodium Phosphate Buffer	Boston Bioproducts	BB-190
33	Sodium Chloride (5 M)	Boston Bioproducts	BM-244
34	Tris-Glycine-Native Running Buffer (10X)	Boston Bioproducts	BP-160
35	Tris-Glycine-SDS Running Buffer (10X)	Boston Bioproducts	BP-150
36	Barbital buffer	Sigma	B5934
37	EDTA (0.5 M, pH 8.0)	Boston Bioproducts	BM-150
38	Bovine serum albumin	Sigma	A2153
39	n-Dodecyl β-D-maltoside	Sigma	D4641
40	n-Octylglucoside	Sigma	10634425001
41	Glycerol	Sigma	G7893
42	Calcium chloride	Boston Bioproducts	MT-140
43	Magnesium chloride	Boston Bioproducts	MT-200
